# Activity-driven myelin sheath growth is mediated by mGluR5

**DOI:** 10.1038/s41593-025-01956-9

**Published:** 2025-05-14

**Authors:** Philipp N. Braaker, Xuelong Mi, Daniel Soong, Jenea M. Bin, Katy Marshall-Phelps, Stephen Bradley, Silvia Benito-Kwiecinski, Julia Meng, Donia Arafa, Claire Richmond, Marcus Keatinge, Guoqiang Yu, Rafael G. Almeida, David A. Lyons

**Affiliations:** 1 Centre for Discovery Brain Sciences, MS Society Edinburgh Centre for Multiple Sclerosis Research, University of Edinburgh, Edinburgh, UK; 2 Bradley Department of Electrical and Computer Engineering, Virginia Polytechnic Institute and State University, Arlington, VA USA; 3 Centre for Clinical Brain Sciences, UK Dementia Research Institute at University of Edinburgh, University of Edinburgh, Edinburgh, UK; 4 Department of Automation, Tsinghua University, Beijing, China

**Keywords:** Glial biology, Myelin biology and repair

## Abstract

Myelination by oligodendrocytes in the central nervous system is influenced by neuronal activity, but the molecular mechanisms by which this occurs have remained unclear. Here we employed pharmacological, genetic, functional imaging and optogenetic-stimulation approaches in zebrafish to assess activity-regulated myelination in vivo. Pharmacological inhibition and activation of metabotropic glutamate receptor 5 (mGluR5) impaired and promoted myelin sheath elongation, respectively, during development, without otherwise affecting the oligodendrocyte lineage. Correspondingly, mGluR5 loss-of-function mutants exhibit impaired myelin growth, while oligodendrocyte-specific mGluR5 gain of function promoted sheath elongation. Functional imaging and optogenetic-stimulation studies revealed that mGluR5 mediates activity-driven high-amplitude Ca^2+^ transients in myelin. Furthermore, we found that long-term stimulation of neuronal activity drives myelin sheath elongation in an mGluR5-dependent manner. Together these data identify mGluR5 as a mediator of the influence of neuronal activity on myelination by oligodendrocytes in vivo, opening up opportunities to assess the functional relevance of activity-regulated myelination.

## Abstract (web summary)

Employing pharmacology, genetics and all-optical approaches in zebrafish, Braaker et al. find that neuronal activity influences the growth of myelin sheaths along axons by signaling through metabotropic glutamate receptor 5 on oligodendrocytes.

## Main

Myelination in the central nervous system (CNS) can be dynamically regulated by a range of extrinsic signals, including neuronal activity^1,2^. In principle, even subtle changes to the number, distribution, length and thickness of myelin sheaths can influence the conduction of action potentials along axons and thus circuit function^3^. Hence, the concept has emerged that activity-driven modulation of myelination may represent a form of nervous system plasticity^4,5^; however, the molecular mechanisms by which neuronal activity influences myelination by oligodendrocytes are not yet clear. One underlying reason for this is the fact that neuronal activity can affect many stages of oligodendrocyte lineage progression (for example refs. ^6–21^), which makes it difficult to experimentally disentangle the mechanisms underlying the influence of active axons on the process of myelination itself. For example, numerous studies have investigated how various neurotransmitter receptors and other activity-related mediators influence myelin, but in all cases where changes to myelin are observed upon manipulation of activity, earlier stages of oligodendrocyte lineage progression are also altered (for example^8,22–36^). Therefore, at present we do not know the identity of the neuronal signals or oligodendrocyte receptors that mediate the effects of neuronal activity on the myelinating behavior per se of oligodendrocytes in vivo.

A range of in vitro, ex vivo and in vivo studies spanning zebrafish, rodents, and humans indicate that the vesicular release of neuronal signals can affect myelination by oligodendrocytes^13,14,37–45^. Indeed, activity-regulated vesicular release of signals from individual axons can affect the number and length of myelin sheaths along axons^37,40,43^, pointing to direct axon–myelin communication. As the principal neurotransmitter of the CNS, glutamate remains a primary candidate driver of axon–myelin signaling^46^; however, to date in vivo*-*based investigations of the roles of ionotropic glutamate receptors in the oligodendrocyte lineage have either revealed roles early in the oligodendrocyte lineage that may influence myelination secondarily as noted above^22,26,27,29^, or limited effects on myelin architecture at all^28,32^. Members of the other main class of glutamate neurotransmitter receptors, metabotropic glutamate receptors (mGluRs), notably mGluR5, are also expressed by oligodendrocytes^47–50^. Metabotropic glutamate receptors represent strong candidate mediators of the effects of activity on myelination, because they can influence cellular signaling and morphology over longer timescales than ionotropic receptors, more in line with observed responses of myelinating cells to activity^51^. Furthermore, mGluRs often converge on localized Ca^2+^ signaling, which is noteworthy given evidence that Ca^2+^ signaling in myelin sheaths is regulated by neuronal activity and can affect myelin sheath fate^52–54^; however, the potential role of mGluRs in mediating activity-regulated myelination in vivo has not yet been investigated.

Here we investigated the role of mGluR5 signaling in myelination in vivo using zebrafish. We found, using pharmacological, gene targeting, functional imaging and optogenetic-stimulation approaches, that mGluR5 mediates myelin sheath growth in response to neuronal activity. This identifies, to our knowledge, the first receptor that mediates the effects of neuronal activity on myelination per se in the intact CNS.

## Results

### mGluR5 manipulation affects myelin sheath length

**Fig. 1:**
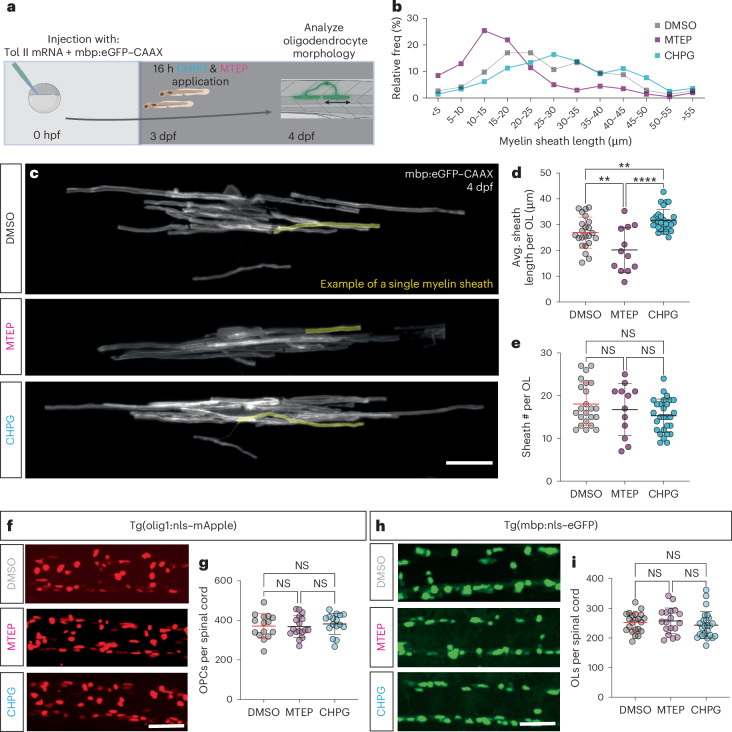
mGluR5 stimulation increases myelin sheath length without affecting cell number. **a**, Protocol to label individual oligodendrocytes, manipulate mGluR5 activity and assess myelination. **b**, Relative frequency of sheath lengths (DMSO *n* = 427, mean 16.79 µm; MTEP *n* = 201, mean 18.41 µm; CHPG *n* = 470, mean 30.44 µm). **c**, Individual oligodendrocytes at 4 dpf, labeled with mbp:eGFP–CAAX, after treatment with DMSO, mGluR5 antagonist MTEP and allosteric agonist CHPG. Scale bar, 15 µm. **d**, Mean sheath length per oligodendrocyte (one OL/fish) post treatment (one-way analysis of variance (ANOVA), *P* < 0.0001; Holm–Šídák’s multiple comparisons test: DMSO versus CHPG *P* = 0.0062, DMSO versus MTEP *P* = 0.0047; CHPG versus MTEP *P* < 0.0001). 1% DMSO (*N* = 23, 26.90 µm ± 6.031), CHPG (*N* = 28, 31.66 µm ± 4.27) and MTEP (*N* = 12, 20.15 µm ± 8.77). **e**, Number of myelin sheaths produced by single oligodendrocytes (one-way ANOVA; *P* = 0.2090, Kruskal–Wallis DMSO versus CHPG *P* = 0.2477; DMSO versus MTEP *P* > 0.9999; CHPG versus MTEP *P* > 0.9999). DMSO, *N* = 23, 18.04 ± 4.995; MTEP, *N* = 12, 16.75 ± 3.89; CHPG *N* = 28, 15.32 ± 6.08). Scale bar, 50 µm. **f**, Representative images of Tg(olig1:nls–mApple) after pharmacological manipulation of mGluR5. Scale bar, 50 µm. **g**, Number OPCs in the spinal cord (one-way ANOVA; *P* = 0.7774; Tukey’s multiple comparisons test, DMSO versus MTEP *P* = 0.9908; DMSO versus CHPG *P* = 0.8579; CHPG versus MTEP *P* = 0.7781), DMSO (*N* = 15); CHPG (*N* = 17); MTEP (*N* = 16). DMSO 371.1 ± 62.39; MTEP 368.5 ± 54.37; CHPG 381.8 ± 53.13. **h**, Representative images of Tg(mbp:nls–eGFP) after pharmacological manipulation of mGluR5. **i**, Number of myelinating oligodendrocytes in the spinal cord (one-way ANOVA; *P* = 0.5155; Tukey’s multiple comparisons test: DMSO versus MTEP *P* = 0.9141; DMSO versus CHPG *P* = 0.7359; CHPG versus MTEP *P* = 0.5037), DMSO (*N* = 22); CHPG (*N* = 25); MTEP (*N* = 18). DMSO, 252 ± 33.24; MTEP, 257.3 ± 43.61; CHPG, 243 ± 45.46. Scale bar, 50 µm. Data indicate mean ± s.d. Source data

Numerous studies have implicated glutamate receptor signaling in myelination. As a precursor to this study, we assessed how a range of chemical compounds targeting different classes of glutamate receptors influenced myelination in larval zebrafish and found that compounds targeting mGluR5 affected myelin sheath length. Therefore, we aimed here to investigate in detail how mGluR5 signaling affects myelination in the CNS. To manipulate mGluR5 signaling, we first treated larval zebrafish with either the mGluR5 antagonist, MTEP (3-((2-methyl-1,3-thiazol-4-yl)ethynyl)pyridine)^55^ or the mGluR5 agonist CHPG ((RS)-2-chloro-5-hydroxyphenylglycine)^56^, from 3 days post-fertilization (dpf) to 4 dpf, shortly after the onset of developmental myelination (Fig. 1a). To visualize myelination, we mosaically expressed the transgene mbp:eGFP–CAAX in individual oligodendrocytes (Fig. 1a)^44^. We found that treatment of animals with MTEP led to a 25% decrease in the length of myelin sheaths relative to controls (Fig. 1b–d). In contrast, treatment with the mGluR5 agonist CHPG increased myelin sheath length by 16% (Fig. 1b–d). We also assessed how these compounds affected the number of myelin sheaths made by individual oligodendrocytes and found that neither MTEP nor CHPG affected sheath number per oligodendrocyte, relative to dimethylsulfoxide (DMSO) controls (Fig. 1e), pointing to a specific effect of mGluR5 signaling on myelin sheath length. Previous studies have implicated glutamate receptor signaling in regulating early stages of oligodendrocyte lineage progression. Therefore, we wanted to assess whether mGluR5 signaling affected oligodendrogenesis, or could instead elicit a specific effect on myelination per se. To test this, we treated animals with MTEP or CHPG between 3 dpf and 4 dpf and assessed whether this altered either OPC (labeled by Tg(olig1:nls–mApple)) or myelinating oligodendrocyte number (labeled by Tg(mbp:nls–eGFP)). We found no changes to the numbers of oligodendrocyte precursor cells (OPCs) or myelinating oligodendrocytes upon treatment with either MTEP or CHPG (Fig. 1f–i), indicating that the effects of these compounds can specifically influence myelin sheath length.

### Genetic loss of mGluR5 function reduces myelin sheath length

**Fig. 2:**
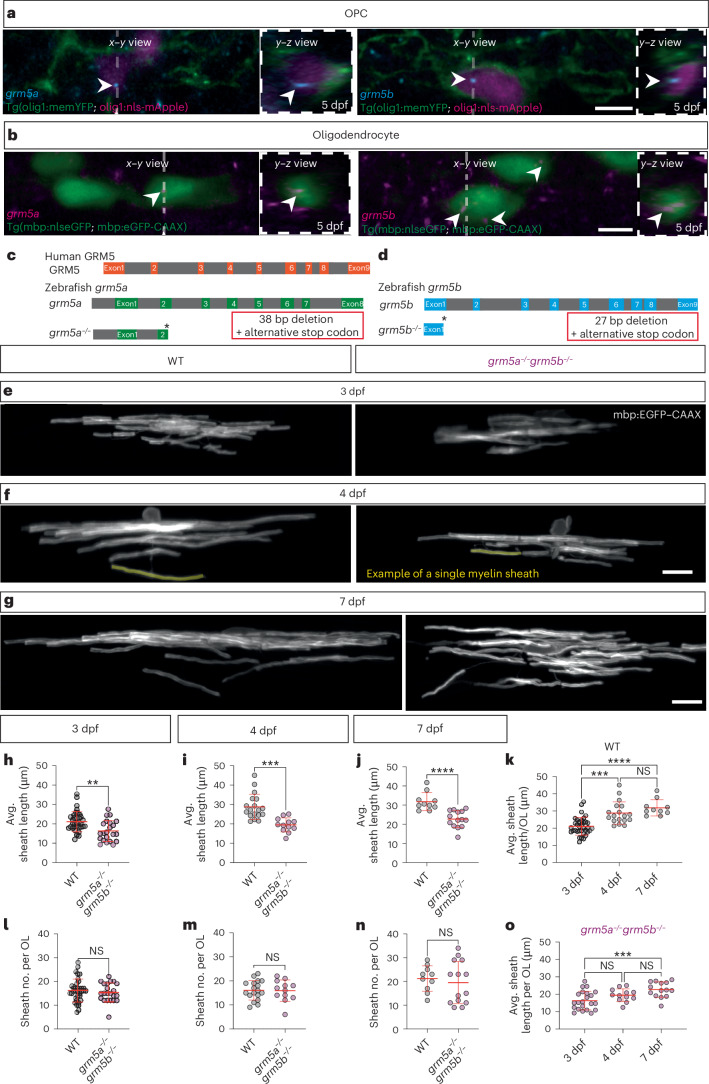
Mutations in genes encoding mGluR5 reduce myelin sheath length without affecting sheath number. **a**, HCR detection of *grm5a* and *grm5b* messenger RNA in OPCs at 5 dpf. Scale bar, 5 μm. **b**: HCR detection of *grm5a* and *grm5b* mRNA in OLs at 5 dpf. Scale bar, 5 μm. **c**,**d**, Schematic showing organization of human and zebrafish genes encoding mGluR5 and their CRISPR/Cas9 based editing that led to identification of mutants with premature STOP codons in exons 2 and 1 of *grm5a* (**c**) and *grm5b* (**d**), respectively. **e**, Images of wild-type (*N* = 35) and *grm5a*^*−/−*^*grm5b*^*−/−*^ (*N* = 22) oligodendrocytes expressing mbp:eGFP–CAAX. **f**, Images of wild-type (*N* = 18) and *grm5a*^*−/−*^*grm5b*^*−/−*^ (*N* = 12) oligodendrocytes expressing mbp:eGFP–CAAX at 4 dpf. **g**, Images of wild-type (*N* = 10) and *grm5a*^*−/−*^*grm5b*^*−/−*^ (*N* = 14) oligodendrocytes expressing mbp:eGFP–CAAX at 7 dpf. Scale bars 15 μm (**e**–**g**). **h**, Mean sheath length of wild-type (*N* = 35, 21.11 ± 5.149) and *grm5a*^*−/−*^*grm5b*^*−/−*^ (*N* = 22, 16.34 ± 5.38) oligodendrocytes, in 3 dpf old animals (two-sided Mann–Whitney *U*-test, *P* = 0.0035). **i**, Mean sheath length of wild-type (*N* = 18, 28.8 ± 6.5) and *grm5a*^*−/−*^*grm5b*^*−/−*^ (*N* = 12, 19.47 ± 3.67) oligodendrocytes, in 4 dpf old animals (two-sided unpaired *t*-test: *P* = 0.0001). **j**, Mean sheath length of wild-type (*N* = 10, 31.79 ± 4.723) and *grm5a*^*−/−*^*grm5b*^*−/−*^ (*N* = 14, 22.68 ± 4.40) oligodendrocytes, in 7 dpf old animals (two-sided unpaired *t*-test: *P* < 0.0001). **k**, Comparison of mean sheath length in wild-type animals between 3, 4 and 7 dpf animals (one-way ANOVA, Kruskal–Wallis test *P* < 0.0001; Dunn’s multiple comparisons test: wild-type 3 dpf versus 4 dpf *P* = 0.0002; wild-type 4 dpf to 7 dpf *P* = 0.7173, wild-type 3 dpf versus 7 dpf *P* < 0.0001). (3 dpf, 21.11 ± 5.149; 4 dpf, 28.8 ± 6.52; 7 dpf, 31.79 ± 4.72). **l**, Number of myelin sheath per oligodendrocyte in 3 dpf wild-type (16.2 ± 4.8) and *grm5a*^*−/−*^*grm5b*^*−/−*^ (15.36 ± 3.94) (two-sided unpaired *t*-test; *P* = 0.4964). **m**, Number of sheaths per oligodendrocyte in 4 dpf wild-type (16.06 ± 4.03) and *grm5a*^*−/−*^*grm5b*^*−/−*^ (15.92 ± 34.46) (two-sided unpaired *t*-test; *P* = 0.9301). **n**, Number of myelin sheath per oligodendrocyte in 7 dpf wild-type (21.2 ± 5.35) and *grm5a*^*−/−*^*grm5b*^*−/−*^ (19.5 ± 8.91) (two-sided unpaired *t*-test; *P* = 0.5971). **o**, Comparison of the mean sheath length in *grm5a*^*−/−*^*grm5b*^*−/−*^ animals between 3, 4 and 7 dpf animals (one-way ANOVA, Kruskal–Wallis test *P* < 0.0001; Dunn’s multiple comparisons test: *grm5a*^*−/−*^*grm5b*^*−/−*^ 3 dpf versus 4 dpf *P* = 0.1675; *grm5a*^*−/−*^*grm5b*^*−/−*^ 4 dpf to 7 dpf *P* = 0.2070, *grm5a*^*−/−*^*grm5b*^*−/−*^ 3 dpf versus 7 dpf *P* < 0.0009) (3 dpf, 16.34 ± 5.38; 4 dpf, 19.47 ± 3.67; 7 dpf, 22.68 ± 4.40). Data show mean ± s.d. Source data

**Extended Data Fig. 1:**
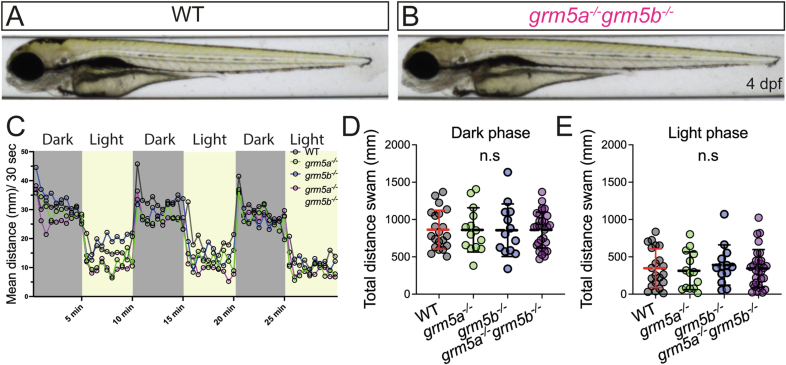
mGluR5 loss of function mutants develop normally and show normal swimming behavior. **A**: Brightfield image of a 4 dpf old zebrafish. **B** Brightfield image of a 4 dpf *grm5a*^−/−^ *grm5b*^−/−^ mutant zebrafish. **C**: Swimming behavior of 5 dpf mGluR5 mutants and WT siblings in light and dark phases (WT N = 21 mean 863.8, SD: ±260.6; *grm5a*^−/−^ N =14, mean: 860.3, SD: ±296.4; *grm5b*^−/−^ N =13, mean: 856.7, SD: ±351.8; *grm5a*^*−/−*^*grm5b*^*−/−*^ N =30, mean:860.3, SD: ±238.9). Average distances swam over time and distinct phases by different groups. **D**: Distance swam by individual fish during all dark phases combined (WT N = 21 mean 863.8, SD: ±260.6; *grm5a*^*-/-*^ N=14, mean: 860.3, SD: ±296.4; *grm5b*^*-/-*^=13, mean: 856.7, SD: ±351.8; *grm5a*^*−/−*^*grm5b*^*-/-*^ N=30, mean:860.3, SD: ±238.9). (One-Way ANOVA; p-value = 0.9999, Tukey’s multiple comparisons test: WT vs. *grm5a*^*-/-*^ p-value = 0.9999, WT vs. *grm5b*^*-/-*^ p-value = 0.9999, WT vs. *grm5a*^*-/-*^*grm5b*^*-/-*^ p-value = 0.9999, *grm5a*^*-/-*^ vs. *grm5b*^*-/-*^ p-value = 0.9999, *grm5a*^*-/-*^ vs. *grm5a*^*-/-*^*grm5b*^*-/-*^ p-value = 0.9999,). **E**: Distance swam by individual fish during all light phases combined (WT N= 21 mean 348, SD: ±259.5; *grm5a*^*-/-*^ N=14, mean: 313.9, SD: ±259.5; *grm5b*^*-/-*^ N=13, mean: 390.8 SD: ±269.3; *grm5a*^*-/-*^*grm5b*^*-/-*^=30, mean:344.5, SD: ±253.3). (One-Way ANOVA; p-value = 0.2334; Tukey’s multiple comparisons test: WT vs. *grm5a*^*-/-*^ p-value = 0.9807, WT vs. *grm5b*^*-/-*^ p-value = 0.9650, WT vs. *grm5a*^*-/-*^*grm5b*^*-/-*^ p-value = 0.9999 *grm5a*^*-/-*^ vs. *grm5b*^*-/-*^ p-value = 0.8653, *grm5a*^*-/-*^ vs. *grm5a*^*-/-*^*grm5b*^*-/-*^ p-value = >0.983, *grm5a*^*-/-*^ vs. *grm5a*^*-/-*^*grm5b*^*-/-*^ p-value = 0.9484). Source data

**Extended Data Fig. 2:**
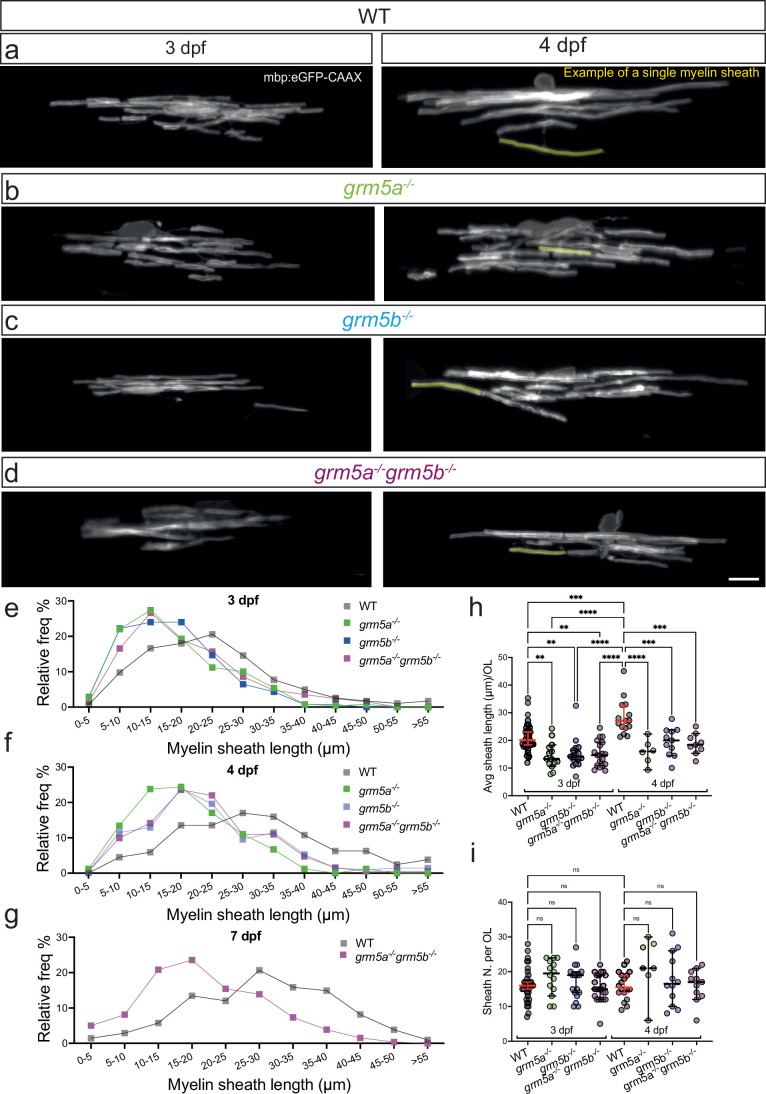
mGluR5 loss of function oligodendrocytes have shorter myelin sheaths and are growing between 3 and 4 dpf. **A**: 3 and 4 dpf wild-type oligodendrocytes labeled with mpb:eGFP-CAAX (N = 36). **B**: 3 and 4 dpf *grm5a*^*-/-*^*grm5b*^*-/-*^ oligodendrocytes at 3 dpf (N = 22). **C**: 3 and 4 dpf *grm5a*^*-/-*^ oligodendrocytes (N = 14). **D**: 3 and 4 dpf *grm5b*^*-/-*^ oligodendrocytes (N = 17). **E:** Relative frequency distribution of individual measured myelin sheath lengths in 3 dpf control and loss of function mutants (WT n = 583, mean: 20.03 µm) (*grm5a*^*-/-*^ n = 259, mean: 14.08 µm) (*grm5b*^*-/-*^ n = 279 mean: 13.48 µm) (*grm5a*^*-/-*^*grm5b*^-/-^ n = 338, mean: 15.89 µm). **F:** Relative frequency distribution of individual measured myelin sheath lengths in 4 dpf control and loss of function mutants (WT n = 288, mean: 27.52 µm; *grm5a*^*-/-*^ n = 164, mean: 15.9 µm; *grm5b*^*-/-*^ n = 209, mean: 19.72 µm; *grm5a*^*-/-*^*grm5b*^*-/-*^ n = 191, mean: 18.88 µm). **G** Relative frequency distribution of individual measured myelin sheath length in 7 dpf WT (n:212 mean: 31.34 µm) and *grm5a*^*-/-*^*grm5b*^*-/-*^ (n = 259, mean: 21.87) animals. **H**: Comparison between sheath lengths per cell in 3 and 4 dpf fish (One-Way ANOVA p-value > 0.0001; Tukey’s multiple comparisons test: 3 dpf WT vs. 3 dpf *grm5a*^*-/-*^ p-value = 0.0038; 3 dpf WT vs. 3 dpf *grm5b*^*-/-*^ p-value = 0.0064; WT 3 dpf vs. 3 dpf *grm5a*^*-/-*^ *grm5b*^*-/-*^p-value = 0.0293; 3 dpf WT vs. 4 dpf WT: p-value = <0.0001; WT 4 dpf vs. 4 dpf *grm5a*^*-/-*^ p-value = <0.0001; WT 4 dpf vs. 4 dpf *grm5b*^*-/-*^ p-value = <0.0028; WT 4 dpf vs. 4 dpf *grm5a*^*-/-*^*grm5b*^*-/-*^ p-value = 0.0002; 3 dpf WT vs 4 dpf *grm5a*^*-/-*^*grm5b*^*-/-*^ p-value = 0.9842; 3 dpf *grm5a*^*-/-*^ vs. 4 dpf WT p-value = <0.0001; 3 dpf *grm5b*^*-/-*^ vs. 4 dps WT p-value = <0.0001, 3 dpf *grm5a*^*-/-*^*grm5b*^*-/-*^ vs. 4 dpf WT p-value = <0.0001). WT 3 dpf mean: 21.11, SD: ±5.15; 3 dpf *grm5a*^*-/-*^ mean= 14.5, SD: ±4.77; 3 dpf *grm5b*^*-/-*^ mean= 15,17 SD: ±5.38; 3 dpf *grm5a*^*-/-*^ *grm5b*^*-/-*^ mean= 16.34 SD: ±5.376; 4 dpf WT mean: 28.8, SD: ±6.519; 4 dpf *grm5a*^*-/-*^ mean: 15.99 SD: ±3.94, 4 dpf *grm5b*^*-/-*^ mean: 20.83 SD: ±6.716; 4 dpf *grm5a*^*-/-*^*grm5b*^*-/-*^ mean: 19.47, SD: ±3.67). **I**: Myelin sheath numbers of 3 and 4 dpf WT and mGluR5 mutants (One-way ANOVA p-value = 0.2457 Tukey’s multiple comparisons test: 3 dpf WT vs. 3 dpf *grm5a*^*-/-*^ p-value = 0.8436; 3 dpf WT vs. 3 dpf *grm5b*^*-/-*^ p-value = 0.9995; WT 3 dpf vs. 3 dpf *grm5a*^*-/-*^ *grm5b*^*-/-*^p-value = 0.9988; 3 dpf WT vs. 4 dpf WT: p-value = >0.9999; WT 4 dpf vs. 4 dpf *grm5a*^*-/-*^ p-value = 0.4523; WT 4 dpf vs. 4 dpf *grm5b*^*-/-*^ p-value = 0.982; WT 4 dpf vs. 4 dpf *grm5a*^*-/-*^*grm5b*^*-/-*^p-value = >0.9999; 3 dpf WT vs. 4 dpf *grm5a*^*-/-*^*grm5b*^*-/-*^ p-value = 0.9992, 3 dpf *grm5a*^*-/-*^ vs. 4 dps WT p-value = 0.8797; 3 dpf *grm5b*^*-/-*^ vs. 4 dps WT p-value = 0.9994, 3 dpf *grm5a*^*-/-*^*grm5b*^*-/-*^ vs. 4 dpf WT p-value = 0.9999). WT 3 dpf mean: 16.2, SD: ±4.80; 3 dpf *grm5a*^*-/-*^ mean: 18.5, SD: ±5.00 3 dpf *grm5b*^*-/-*^ mean: 17, SD: ±4.74; 3 dpf *grm5a*^*-/-*^ *grm5b*^*-/-*^ mean: 15.36 SD: ±3.94; 4 dpf WT mean: 16.06 SD: ±4.04; 4 dpf *grm5a*^*-/-*^ mean: 20.5 SD: ±8.23, 4 dpf *grm5b*^*-/-*^ mean: 17.83 SD: ±7.49; 4 dpf *grm5a*^*-/-*^*grm5b*^*-/-*^ mean: 15.92, SD: ±4.46). Source data

Given evidence that pharmacological manipulation of mGluR5 affected myelination, we wanted to assess the expression and function of genes encoding endogenous mGluR5 in zebrafish. As a result of the partial genome duplication in zebrafish^57^, mGluR5 is encoded by two genes, *grm5a* and *grm5b*. Using in situ hybridization chain reaction (HCR) we observed localization of *grm5a* and *grm5b* in the oligodendrocyte lineage (Methods and Fig. 2a,b). To test whether endogenous mGluR5 affects myelination, we established mutants that disrupt mGluR5 using CRISPR-Cas9 (Methods) and identified mutations that resulted in premature stop codons in the open reading frames of both *grm5a* and *grm5b* (Fig. 2c,d). Both single- and double-mutant animals exhibit grossly normal early development and behavior (Extended Data Fig. 1a–e), and are viable and fertile as adults. To assess myelination, we repeated our single cell mosaic labeling strategy and assessed the number and length of myelin sheaths made by *grm5a*^*−/−*^*grm5b*^*−/−*^ double-mutant oligodendrocytes at 4 dpf (Fig. 2f). Similar to our pharmacological inhibition of mGluR5 using MTEP, we observed a reduction in myelin sheath length in both single mutants (Extended Data Fig. 2) as well as in *grm5a*^*−/−*^*grm5b*^−/−^ double mutants (Fig. 2i and Extended Data Fig. 2), without any effect observed on the number of myelin sheaths made per oligodendrocyte (Fig. 2l–n and Extended Data Fig. 2).

**Extended Data Fig. 3:**
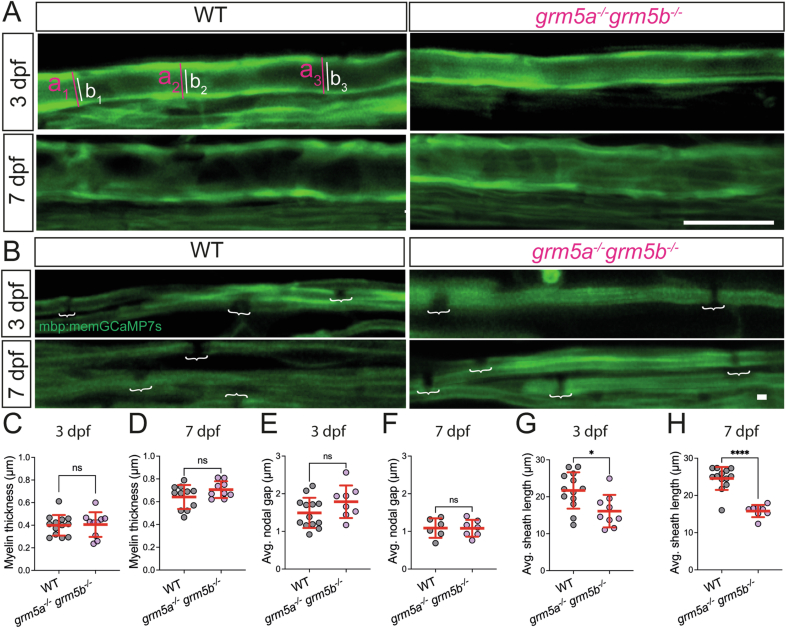
mGluR5 loss of function mutation does not alter myelin thickness, or nodal gap width. **A:** Confocal images of mbp:memGCaMP7s used to assess myelin thickness around the Mauthner axon in wild-type and *grm5a*^*-/-*^ *grm5b*^*-/-*^ at 3 and 7 dpf. **B:** Confocal images of mbp:memGCaMP7s indicating myelin sheaths and nodal gaps in wild-type and *grm5a*^*-/-*^ *grm5b*^*-/-*^ animals at 3 and 7 dpf. **C:** Myelin thickness in wild-type (N = 13, mean: 0.40 µm, SD: ±0.09) and *grm5a*^*-/-*^ *grm5b*^*-/-*^ (N = 10, mean: 0.41 µm, SD: ±0.11) animals at 3 dpf (Unpaired t-test: p-value = 0.8754). **D:** Myelin thickness in wild-type (N = 11, mean: 0.64 µm, SD: ±0.11) and *grm5a*^*-/-*^ *grm5b*^*-/-*^ (N = 9, mean: 0.71 µm, SD: ±0.07) animals at 7 dpf (Unpaired t-test: p-value = 0.1385). **E:** Avg. nodal gap per animal in wild-type (N = 13, mean: 1.49 µm, SD: ±0.40) and *grm5a*^*-/-*^ *grm5b*^*-/-*^ animals (N = 9, mean: 1.80 µm, SD: ±0.41) at 3 dpf (Unpaired t-test: p-value = 0.091). **F:** Avg. nodal gap per animal in wild-type (N = 6, mean: 1.09 µm, SD: ±0.26) and *grm5a*^*-/-*^ *grm5b*^*-/-*^ (N = 7, mean: 1.08 µm, SD: ±0.22) animals at 7 dpf (Unpaired t-test: p-value = 0.9618). **G:** Avg. Myelin sheath length per animal in wild-type (N = 13, mean: 21.71 µm SD: ±4.92) and *grm5a*^*-/-*^ *grm5b*^*-/-*^ (N = 9, mean: 16.11 µm SD: ±4.40) animals at 3 dpf (Unpaired t-test: p-value = 0.0128). **H:** Avg. Myelin sheath length per animal in wild-type (N = 13, mean: 24.57 µm, SD: ±3.08) and *grm5a*^*-/-*^ *grm5b*^*-/-*^ (N = 8 mean:15.81 µm, SD: ±1.64) animals at 7 dpf (Mann–Whitney test: p-value = 0.0002). Source data

**Extended Data Fig. 4:**
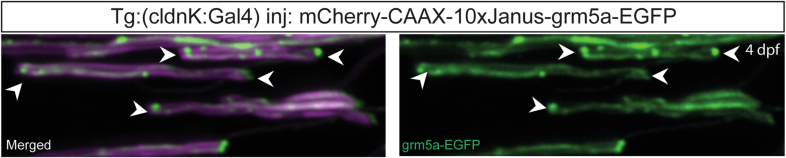
mGluR5 can localize to the tips of myelin sheaths. Subcellular localization of grm5a-eGFP in myelin sheaths (mCherry-CAAX). Arrows indicating grm5a-eGFP along myelin sheaths with aggregates at the growing tips. Scale bar, 10 μm.

To test whether these phenotypes reflected impaired elongation of myelin sheaths or a shrinkage of myelin sheaths by 4 dpf, we looked at an earlier stage (3 dpf) and found that *grm5a*^*−/−*^, *grm5b*^*−/−*^, and *grm5a*^−/−^ *grm5b*^−/−^ double mutants also all had reduced sheath lengths compared to controls. This shows that myelin sheaths do not shrink over time in *grm5* mutants, but that they have an impaired ability to elongate (Fig. 2e,h and Extended Data Fig. 2). To test the longer-term effect of mGluR5 loss of function on sheath growth, we examined oligodendrocytes at a later stage of development at 7 dpf in *grm5a*^*−/−*^*grm5b*^*−/−*^ double mutants. Again, we observed that myelin sheath length, but not sheath number, was reduced relative to controls, indicating that myelin sheaths do not recover to normal lengths over this period of time (Fig. 2g,j). In controls sheath lengths increased by 27% between 3 and 4 dpf and by 9.5% between 4 and 7 dpf (Fig. 2k). Notably, in *grm5a*^*−/−*^*grm5b*^*−/−*^ double mutants, sheath lengths changed by 17% between 3 and 4 dpf and by 15% between 4 and 7 dpf (Fig. 2o), indicating that sheath growth can occur over time in animals with loss of mGluR5 function, but that the growth rate is reduced at early stages of sheath elongation. Over this time-period (3–7 dpf) we did not detect any change in myelin thickness or the width of nodal gaps between adjacent myelin sheaths in mutant animals (Extended Data Figs. 3 and 4), but cannot rule out potential effects at later stages or on organization along individual myelinated axons. Together our data indicate that the principal effect of mGluR5 loss of function is on myelin sheath elongation, soon after their formation.

### mGluR5 in oligodendrocytes drives sheath elongation

**Fig. 3:**
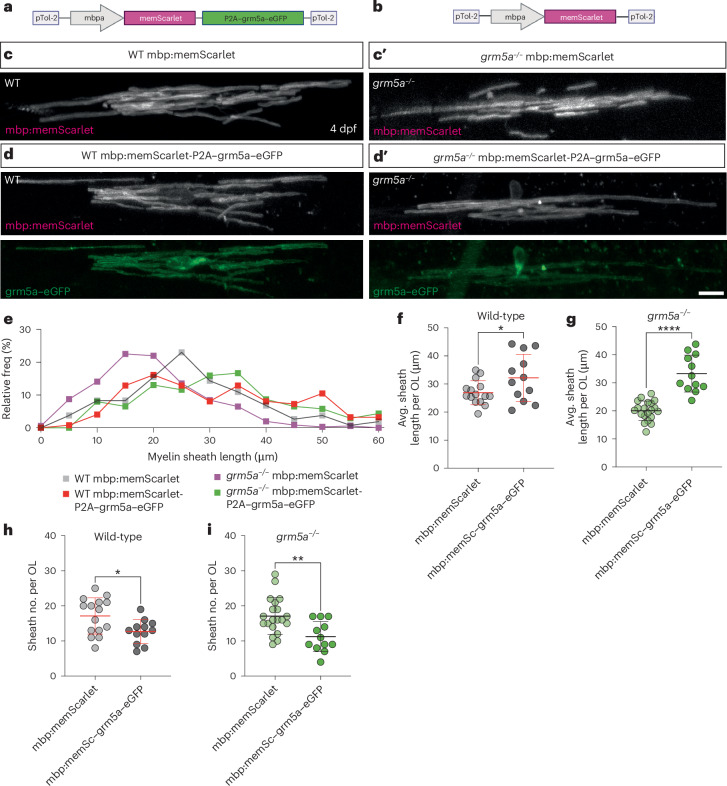
Oligodendrocyte-restricted expression of *grm5a* promotes myelin sheath elongation. **a**, Construct used to express mGluR5 tethered to eGFP, alongside a membrane anchored reporter mScarlet, in myelinating oligodendrocytes. **b**, Control construct used to express fluorescent reporter in myelinating oligodendrocytes. **c**,**c**′, Images of oligodendrocytes expressing control mbp:memScarlet in wild-type (WT) (**c**) (*N* = 15) and *grm5a*^*−/−*^ (**c′**) (*N* = 20) animals at 4 dpf. **d**,**d**′, Oligodendrocytes expressing mbp:memScarlet-P2A–grm5a–eGFP in WT (**d**) (*N* = 12) and *grm5a*^*−/−*^ (**d**′) (*N* = 12) animals at 4 dpf. Scale bar, 10 μm. **e**, Relative frequency distribution of individual myelin sheath lengths. WT mbp:memScarlet *n* = 265 (mean 26.49 µm); WT mbp:memScarlet-P2A–grm5a–eGFP *n* = 124 (mean 31.55 µm); *grm5a*^*−/−*^ mbp:memScarlet *n* = 365 (mean 19.54 µm); *grm5a*^*−/−*^ mbp:memScarlet-P2A–grm5a–eGFP *n* = 138 (mean 31.58 µm). **f**, Mean sheath length of WT oligodendrocytes expressing mbp:memScarlet-P2A–grm5a–eGFP (*N* = 12, 32.19 ± 8.41) or mbp:memScarlet (*N* = 15, 26.92 ± 4.28) (two-sided unpaired *t*-test, *P* = 0.0445). **g**, Mean myelin sheath length of *grm5a*^*−/−*^ oligodendrocytes expressing mbp:memScarlet-P2A–grm5a–eGFP (*N* = 12, 33.25 ± 6.57) or mbp:memScarlet (*N* = 20, 20.05 ± 3.45) (two-sided unpaired *t*-test, *P* = <0.0001). **h**, Sheath number per oligodendrocytes expressing mbp:memScarlet-P2A–grm5a–eGFP (12.67 ± 3.46) or mbp:memScarlet (17.13 ± 5.17) (two-sided unpaired *t*-test, *P* = 0.0164). **i**, Sheath number per oligodendrocytes in *grm5a*^*−/−*^ mutants expressing mbp:memScarlet-P2A–grm5a–eGFP (*N* = 12, 11.25 ± 4.33) or mbp:memScarlet (*N* = 20, 17.1 ± 5.21) (two-sided unpaired *t*-test, *P* = 0.0027). Scale bar, 10 μm. Data show mean ± s.d. Source data

**Extended Data Fig. 5:**
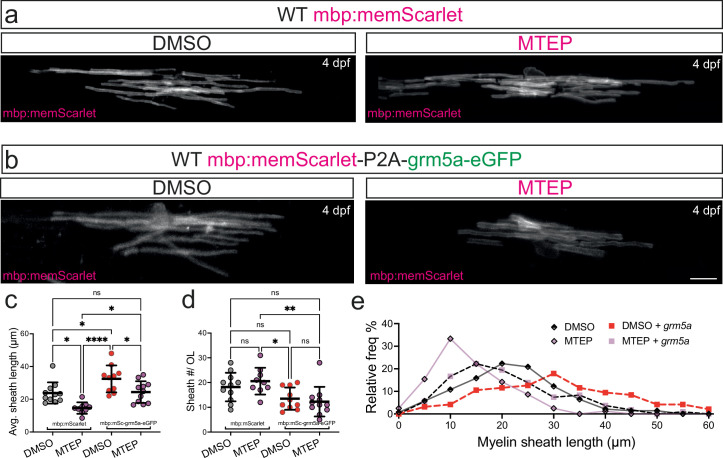
The effects of oligodendrocyte-specific expression of grm5a is abrogated by MTEP. **A:** Confocal images of individual oligodendrocytes in WT larvae expressing mbp:memScarlet treated with DMSO (N = 11) and MTEP at 4 dpf (N = 9). **B:** Confocal images of individual oligodendrocytes in WT larvae expressing mbp:memScarlet-P2A-grm5a-eGFP treated with DMSO (N = 10) and MTEP at 4 dpf (N = 11). **C:** Avg. myelin sheath length in DMSO and MTEP-treated animals expressing mbp:memScarlet or mbp:memScarlet-P2A-grm5a-eGFP (One-Way ANOVA: <0.0001; Tukey’s multiple comparisons test; mbp:memScarlet DMSO vs. mbp:memScarlet MTEP p-value = 0.0163; mbp:memScarlet DMSO vs. mbp:memScarlet-P2A-grm5a-eGFP DMSO p-value = 0.0221; mbp:memScarlet-P2A-grm5a-eGFP DMSO vs. mbp:memScarlet-P2A-grm5a-eGFP MTEP p-value = 0.0351; mbp:memScarlet DMSO; mbp:memScarlet-P2A-grm5a-eGFP MTEP p-value = 0.9974; mbp:memScarlet MTEP vs. mbp:memScarlet-P2A-grm5a-eGFP MTEP p-value = 0.0101; mbp:memScarlet MTEP vs. mbp:memScarlet-P2A-grm5a-eGFP DMSO p-value = <0.0001). DMSO mbp:memScarlet-P2A-grm5a-eGFP mean: 32.46, SD: ±8.232; MTEP mbp:memScarlet-P2A-grm5a-eGFP mean: 24.39 SD: ±6.62, DMSO mbp:memScarlet mean: 23.85 SD: ±6.46; MTEP mbp:memScarlet mean: 14.65, SD: ±3.51). **D:** Myelin sheath number in DMSO and MTEP-treated animals expressing mbp:memScarlet or mbp:memScarlet-P2A-grm5a-eGFP (One-Way ANOVA p-value = 0.0049; Tukey’s multiple comparisons test; mbp:memScarlet DMSO vs. mbp:memScarlet MTEP p-value = 0.7695; mbp:memScarlet DMSO vs. mbp:memScarlet-P2A-grm5a-eGFP DMSO p-value = 0.2217; mbp:memScarlet-P2A-grm5a-eGFP DMSO vs. mbp:memScarlet-P2A-grm5a-eGFP MTEP p-value = 0.9553; mbp:memScarlet DMSO vs. mbp:memScarlet-P2A-grm5a-eGFP MTEP p-value = 0.0710; mbp:memScarlet MTEP vs. mbp:memScarlet-P2A-grm5a-eGFP MTEP p-value = 0.0092; mbp:memScarlet MTEP vs. mbp:memScarlet-P2A-grm5a-eGFP DMSO p-value = 0.0379). DMSO mbp:memScarlet-P2A-grm5a-eGFP mean: 13.5, SD: ±4.478; MTEP mbp:memScarlet-P2A-grm5a-eGFP mean: 12.27, SD: ±5.99, DMSO mbp:memScarlet mean: 18.18, SD: ±5.78; MTEP mbp:memScarlet mean: 20.56, SD: ±5.41). **E:** Relative frequency distribution of individual sheath length (n = 504) DMSO mbp:memScarlet-P2A-grm5a-eGFP n = 95, mean: 30.54; MTEP mbp:memScarlet-P2A-grm5a-eGFP n = 108, mean: 20.62; DMSO mbp:memScarlet n = 139, mean:21.7; MTEP mbp:memScarlet n = 162, mean: 13.64). Source data

Our pharmacological and mutant analysis point to a role for mGluR5 in regulating myelination, but cannot distinguish whether this reflects a function in oligodendrocytes or in other cells, for example neurons or astrocytes, which are known to express mGluR5, reflecting broader roles^58^ that could influence myelination indirectly. To test whether mGluR5 functions in oligodendrocytes to regulate myelin sheath growth, we expressed full-length grm5a fused with enhanced green fluorescent protein (eGFP) or grm5b fused with eGFP in oligodendrocytes using the myelin basic protein *(mbp)* promoter (Fig. 3a,d), both together with mbp:memScarlet to study oligodendrocyte morphology (Fig. 3b,c and Methods). We prioritized the use of grm5a–eGFP for our interventional studies as its expression under the control of the *mbp* promoter led to consistent fusion protein expression, as noted in separate transgenic labeling strategies (Extended Data Fig. 4). Myelinating oligodendrocyte-specific grm5a–eGFP expression in wild-type animals increased myelin sheath length compared to controls at 4 dpf, indicating a gain of function (Fig. 3d–f,j). Of note, this increase in myelin sheath length was accompanied by a reduction in myelin sheath number, suggesting that strongly promoting myelin sheath elongation may reduce sheath number (Fig. 3h,j and Discussion). Therefore, we next tested whether expression of grm5a in myelinating oligodendrocytes is sufficient to rescue the reduction of myelin sheath length observed in *grm5a*^*−/−*^ mutants. Indeed, we saw that transgenic expression of grm5a–eGFP in oligodendrocytes rescues the myelin sheath length deficits of *grm5a*^*−/−*^ mutants (Fig. 3d,e,g). Similar to observations in wild-type animals, this promotion of sheath elongation was associated with a reduction in myelin sheath number per oligodendrocyte (Fig. 3i,j). To independently test that the effect of this construct on myelination was mediated by functional mGluR5, we carried out the myelinating oligodendrocyte-specific analyses in animals treated with MTEP. These analyses showed that the effect of grm5a–eGFP expression in oligodendrocytes was reduced in the presence of mGluR5 inhibition (Extended Data Fig. 5), further corroborating the premise that mGluR5 functions autonomously in oligodendrocytes to drive myelin sheath elongation.

Given that mGluR5 signaling converges on Ca^2+^ activity in other contexts, and that we and others have linked Ca^2+^ activity in myelin to sheath elongation^52,53^, we next wanted to test whether mGluR5 activation affected myelin Ca^2+^ activity.

### mGluR5 influences Ca^2+^ activity in myelin sheaths

Previous studies have shown that localized Ca^2+^ activity can be observed within myelin sheaths^59,60^ and that this affects the fate and growth of sheaths^52,53^. In addition, mGluR5 signaling in other cell types such as neurons and astrocytes, can converge on various Ca^2+^ signaling pathways. Therefore, we wanted to investigate to what extent manipulation of mGluR5 signaling influences Ca^2+^ activity in myelinating oligodendrocytes. To visualize myelin Ca^2+^ activity we generated a transgenic line in which the genetically encoded calcium indicator GCaMP7s is tethered to the membrane and expressed under the control of the myelin basic protein promoter (Methods). To deepen our understanding of how mGluR5 influences myelin sheath elongation, we wanted to test whether mGluR5 could directly affect myelin Ca^2+^ activity.

**Extended Data Fig. 6:**
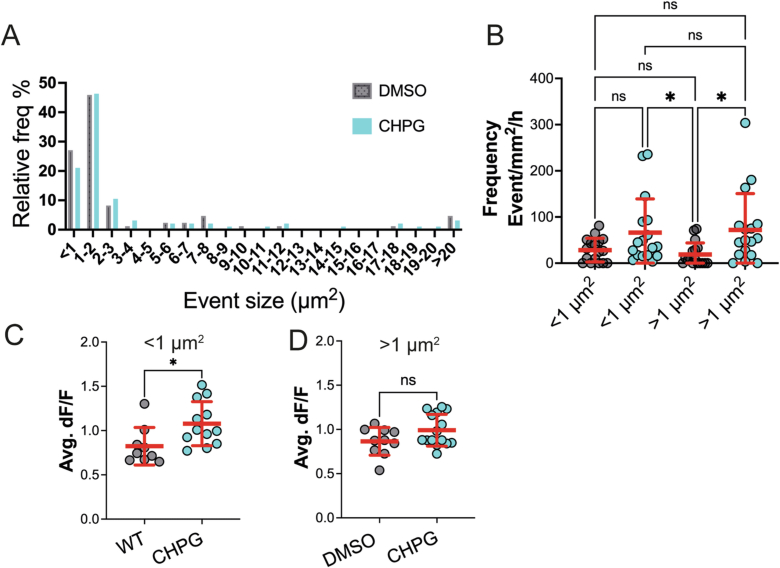
AQuA2 detects small and large myelin Ca^2+^ transients that are mGluR5-regulated. **A**: Relative frequency distribution of myelin Ca^2+^ transients of different sizes (1 µm^2^ bins), after 1 h CHPG and DMSO treatment (DMSO n = 85; CHPG n = 95). **B**: Frequency of myelin Ca^2+^ transients after 1 h of CHPG treatment separated by size (less than or greater than 1 µm^2^) (DMSO N = 17; CHPG N = 17) (One-way Anova p-value = 0.0084; Dunn’s multiple comparisons test; DMSO < 1 µm^2^ vs. < 1 µm^2^ CHPG p-value = >0.9999; DMSO < 1 µm^2^ vs. CHPG < 1 µm^2^ p-value = 0.5854; DMSO < 1 µm^2^ vs. > 1 µm^2^ CHPG p-value = 0.3726; DMSO > 1 µm^2^ vs. > 1 µm^2^ CHPG p-value = 0.0384; DMSO < 1 µm^2^ vs. > 1 µm^2^ CHPG p-value = 0.0200; CHPG < 1 µm^2^ vs. > 1 µm^2^ CHPG p-value = >0.9999). DMSO < 1 µm^2^ mean: 28.3, SD: ±25.71; DMSO > 1 µm^2^ mean: 18.69 SD: ±25.11; CHPG < 1 µm^2^ mean: 66.06, SD: ±73.09; CHPG > 1 µm^2^ mean: 71.96, SD: ±78.67). **C**: Avg. Ca^2+^ transients amplitude comparison of small ( < 1 µm^2^) Ca^2+^ transients after 1 h treatment with DMSO (N = 9, mean: 0.82, SD: ±0.21) and CHPG (N = 12, mean: 1.08, SD: ±0.25) (Mann–Whitney test; p-value = 0.0148). **D**: Comparison of large ( > 1 µm^2^) Ca^2+^ transients amplitude after 1 h treatment with DMSO (mean: 0.87, SD: ±0.16) and CHPG (mean: 0.99, SD: ±0.18) (Unpaired t-test; p-value = 0.0853). Source data

To test how mGluR5 affected myelin Ca^2+^ activity, we co-treated animals with the mGluR5 agonist CHPG and MS222 (Tricaine) a well-characterized blocker of Na^+^ channels in zebrafish. This provided a background of reduced network activity, to enrich for the possibility of identifying an mGluR5 agonist-specific effect on myelin Ca^2+^ activity. To assess Ca^2+^ transients in myelin, we used an automated machine learning based pipeline, AQuA2, which allows automated unbiased detection of Ca^2+^ events of different sizes, without preselection of specific regions of interest (Methods and Extended Data Fig. 6)^61^.

**Fig. 4:**
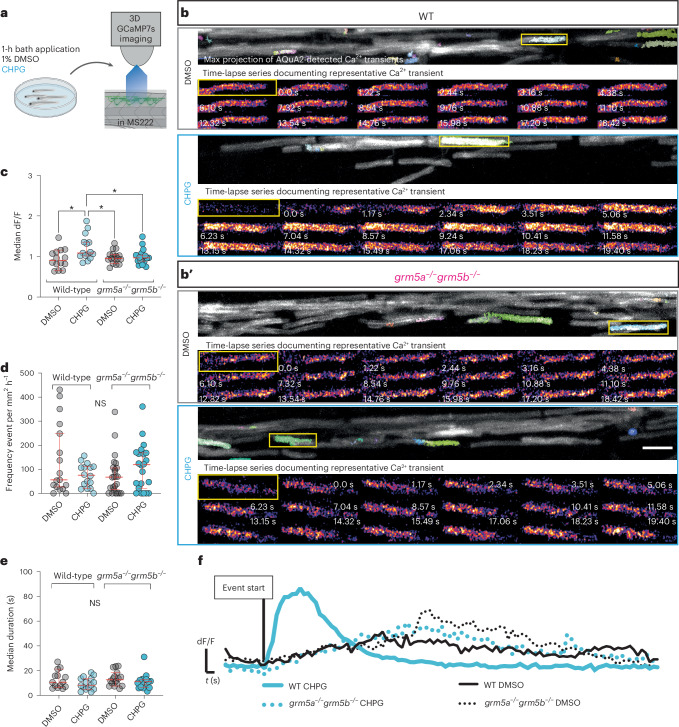
Endogenous mGluR5 is required for CHPG-induced high-amplitude myelin Ca^2+^ transients to occur. **a**, Schematic of treatment of larvae and live imaging of Ca^2+^ activity in myelin sheaths. **b**,**b′**, Top shows representative maximum time series projections of Tg(mbp:memGCaMP7s) fluorescence with Ca^2+^ transients detected by AQuA2 false-colored automatically; bottom show single time-point images from the time-lapse series and highlight transients identified within yellow boxes. WT (*N* = 14) (**b**) and *grm5a*^−/−^*grm5b*^−/−^ animals (**b′**) (*N* = 16) treated with vehicle and CHPG (WT *N* = 15; *grm5a*^−/−^*grm5b*^−/−^ *N* = 17). Scale bar, 10 µm. **c**, Median amplitude (dF/F) of Ca^2+^ transients per fish (one-way ANOVA; *P* = 0.0079; Tukey’s multiple comparison test; WT DMSO versus CHPG *P* = 0.111, WT DMSO versus *grm5a*^−/−^*grm5b*^−/−^ DMSO *P* = 0.9633; WT DMSO versus *grm5a*^*−/−*^*grm5b*^*−/−*^ CHPG *P* = 0.9210, WT CHPG versus *grm5a*^*−/−*^*grm5b*^*−/−*^ DMSO *P* = 0.0310, WT CHPG versus *grm5a*^*−/−*^*grm5b*^*−/−*^ CHPG *P* = 0.0407; *grm5a*^*−/−*^*grm5b*^−/−^ CHPG versus *grm5a*^*−/−*^*grm5b*^*−/−*^ DMSO *P* = 0.9987) (WT DMSO *N* = 14, 0.94 ± 0.24, WT CHPG *N* = 15, 1.21 ± 0.31, *grm5a*^*−/−*^*grm5b*^*−/−*^ DMSO *N* = 16, 0.98 ± 0.16, *grm5a*^*−/−*^*grm5b*^*−/−*^ CHPG *N* = 17, 0.99 ± 0.19). **d**, Frequency of myelin Ca^2+^ transients in CHPG and DMSO-treated *grm5a*^*−/−*^*grm5b*^*−/−*^ mutant and WT animals (one-way ANOVA *P* = 0.1658; Holm–Šídák’s multiple comparisons tests: WT DMSO versus WT CHPG *P* = 0.3022; WT DMSO versus *grm5a*^*−/−*^*grm5b*^*−/−*^ DMSO *P* = 0.2591; WT CHPG versus *grm5a*^*−/−*^*grm5b*^*−/−*^ DMSO *P* = 0.9840; WT CHPG versus *grm5a*^*−/−*^*grm5b*^*−/−*^ CHPG *P* = 0.6868; WT DMSO versus *grm5a*^*−/−*^*grm5b*^*−/−*^ CHPG *P* = 0.6868). WT DMSO 138 ± 149.2, WT CHPG 74.39 ± 48.68, *grm5a*^*−/−*^*grm5b*^*-/*^, DMSO 75.04 ± 82.95, *grm5a*^*−/−*^*grm5b*^*−/−*^ CHPG 108.9 ± 95.43. Scale bar, 10 µm. **e**, Median duration of Ca^2+^ transients per fish (one-way ANOVA *P* = 0.2687; Tukey’s multiple comparisons test: WT DMSO versus WT CHPG *P* = 0.4745; WT DMSO versus *grm5a*^*−/−*^*grm5b*^*−/−*^ DMSO *P* = 0.9864; WT DMSO versus *grm5a*^*−/−*^*grm5b*^*−/−*^ CHPG *P* = 0.8633; WT CHPG versus *grm5a*^*−/−*^*grm5b*^*−/−*^ DMSO *P* = 0.2638, WT CHPG versus *grm5a*^*−/−*^*grm5b*^*−/−*^ CHPG *P* = 0.08864; *grm5a*^*−/−*^*grm5b*^*−/−*^ DMSO versus *grm5a*^*−/−*^*grm5b*^*−/−*^ CHPG *P* = 0.6516). WT DMSO 12.76 ± 6.964, WT CHPG 9.495 ± 5.044, *grm5a*^*−/−*^*grm5b*^*−/−*^, DMSO 13.52 ± 5.936, *grm5a*^*−/−*^*grm5b*^*−/−*^, CHPG 11.06 ± 6.233. **f**, Ca^2+^ transient dF/F traces of the events shown in corresponding conditions in **b** and **b'**. Data show mean ± s.d. Source data

**Extended Data Fig. 7:**
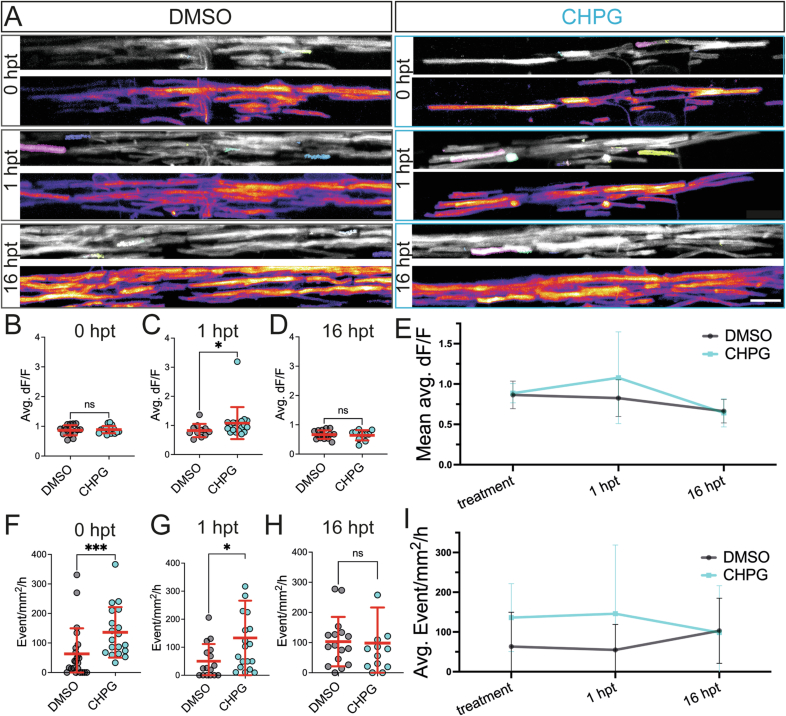
CHPG treatment affects myelin Ca^2+^ transients. **A:** Representative time-lapse images with AQuA2-detected transient shown (top) and Fire LUT (bottom) of mbp:mem:GCaMP7s expressing oligodendrocytes treated with DMSO and CHPG after immediate application of compounds (top rows), 1 h (middle rows) and 16 h (bottom rows) after drug application (hpt). Scale bar, 10 µm. **B:** Avg. dF/F of myelin Ca^2+^ transients immediately after CHPG application (0 hpt) (DMSO N = 16, mean: 0.87, SD: ±0.17; CHPG N = 19 mean: 0.87, SD: ±0.12) (unpaired t-test: p-value = 0.6774). **C:** Avg. dF/F of myelin Ca^2+^ transients 1 h after CHPG application (1 hpt) (DMSO N = 16, mean: 0.82, SD: ±0.23; CHPG N = 19, mean: 1.08, SD: ±0.55) (Mann–Whitney test: p-value = 0.0275). **D:** Avg. dF/F of myelin Ca^2+^ transients 16 h after CHPG application (16 hpt) (DMSO N = 15 mean: 0.66, SD: ±0.15; CHPG N = 10 mean: 0.64, SD: ±0.17) (unpaired t-test: p-value = 0.7351). **E:** Mean of the average dF/F of myelin Ca^2+^ transients after 0, 1 and 16 hpt after CHPG/DMSO application. **F:** Frequency of myelin Ca^2+^ transients per animal immediate after CHPG application (0 hpt) (DMSO N = 23, mean: 63.2 SD: ±86.53; CHPG N = 19, mean: 136, SD: ±85.49) (Mann–Whitney test: p-value = 0.0005). **G:** Frequency of myelin Ca^2+^ transients per animal 1 h after CHPG application (1 hpt) (DMSO = 16, mean: 50.15, SD: ±61.26; CHPG N = 19, mean: 133.1, SD: ±133.0) (Mann–Whitney test: p-value = 0.0159). **H:** Frequency of myelin Ca^2+^ transients per animal 16 h after CHPG application (16 hpt) (DMSO N = 16, mean: 103.1, SD: ±81.94; CHPG N = 13 mean: 98.40, SD: ±118.1) (Mann–Whitney test: p-value = 0.3980). **I**: Mean Frequency of myelin Ca^2+^ transients over treatment, 1 hpt and 16 hpt. Source data

**Extended Data Fig. 8:**
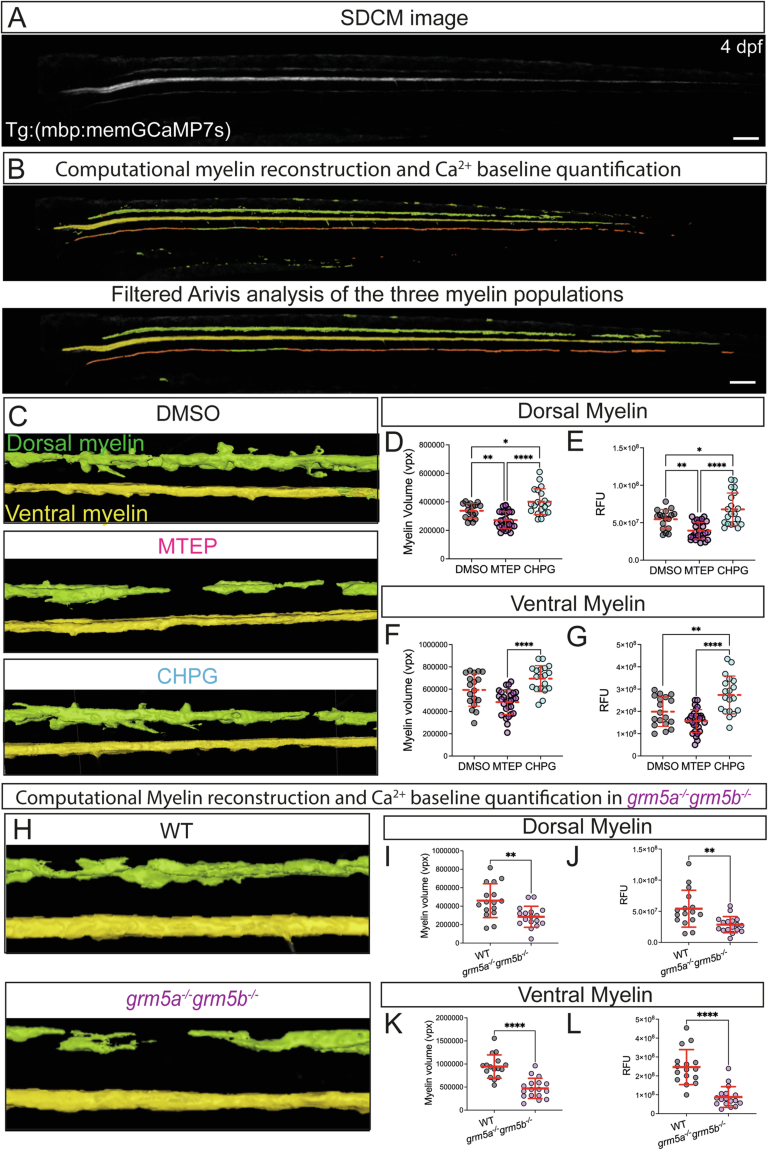
mGluR5 regulates myelin Ca^2+^ levels and myelin volume. Whole CNS measurement of mbp:memGCaMP7s fluorescent intensity and myelin volume using computational 3D reconstruction of SDCM images. **A**: Representative image of a 4 dpf zebrafish expressing mbp:memGCaMP7s in spinal oligodendrocytes and Schwann cells. Scale bar,100 µm. **B**: Computational reconstruction of the transgenic myelin reporter mbp:memGCaMP7s used to assess myelin Ca^2+^ baseline activity and myelin volume. **C**: Reconstruction of 4 dpf CNS myelin in animals treated with DMSO (N = 17), MTEP (N = 28) and CHPG (N = 20). Green reflects myelin along dorsal axons, while yellow labels ventral myelin. **D**: Doral myelin as volumetric pixel volume (vpx) (One-was ANOVA p-value = <0.0001; Tukey’s multiple Comparisons test: DMSO vs. CHPG p-value = 0.0194; DMSO vs. MTEP p-value = 0.0090; CHPG vs. MTEP p-value = <0.0001) DMSO: mean: 337125, SD: ±51246; MTEP mean: 271853, SD: ±60709; CHPG mean: 400665, SD: ±90675). **E**: Relative Fluorescent intensity (RFU) in dorsal myelin sheaths of mbp:memGCaMP7s, reflecting baseline myelin Ca^2+^ activity (One-was ANOVA p-value = <0.0001; Tukey’s multiple Comparisons test: DMSO vs. CHPG p-value = 0.0344; DMSO vs. MTEP p-value = 0.0068; CHPG vs. MTEP p-value = <0.0001; DMSO: mean: 54580775 SD: ±13046833; MTEP mean: 39357143, SD: ±21823431; CHPG mean: 67789985, SD: ±21823431). **F**: Ventral myelin as volumetric pixel volume (vpx) (One-was ANOVA p-value = <0.0001; Tukey’s multiple Comparisons test: DMSO vs. CHPG p-value = 0.0398; DMSO vs. MTEP p-value = 0.0131; CHPG vs. MTEP p-value = <0.0001). DMSO: mean: 593429, SD: ±146357; MTEP mean: 484280, SD: ±111349; CHPG mean: 694801, SD: ±116964). **G**: Relative Fluorescent intensity (RFU) in ventral myelin sheaths of mbp:memGCaMP7s, reflecting baseline myelin Ca^2+^ activity (One-was ANOVA p-value = <0.0001; Tukey’s multiple Comparisons test: DMSO vs. CHPG p-value = 0.0030; DMSO vs. MTEP p-value = 0.1009; CHPG vs. MTEP p-value = <0.0001). DMSO: mean: 198536209, SD: ±66337105; MTEP mean: 156607143, SD: ±50820920; CHPG mean: 273645670, SD: ±84901986). **H**: Representative reconstruction of wild-type (N = 16) and *grm5a*^*-/-*^ *grm5b*^*-/-*^ (N = 17) expression of mbp:memGCaMP7s reporting dorsal (green) and Ventral axon myelination (yellow). **I**: Doral myelin volume in of wild-type and *grm5a*^*-/-*^ *grm5b*^*-/-*^ animals as volumetric pixel volume (vpx) (Unpaired t-test p-value = 0.0024). **J**: Relative Fluorescent intensity (RFU) in dorsal myelin sheaths of wild-type (N = 16, mean: 54180897, SD: ±29591313 and *grm5a*^*-/-*^ *grm5b*^*-/-*^ (N = 17, mean: 28549095, SD: ±13030576) animals expressing mbp:memGCaMP7s, reflecting baseline myelin Ca^2+^ activity (Unpaired t-test p-value = 0.0027). **K**: Ventral myelin volume in of wild-type (N = 16, mean: 944652 SD: ±252246) and *grm5a*^*-/-*^ *grm5b*^*-/-*^ (N = 17, mean: 470384, SD: ±219208) animals as volumetric pixel volume (vpx) (Unpaired t-test p-value = <0.0001). **L**: Relative Fluorescent intensity (RFU) in ventral myelin sheaths of wild-type (N = 16, mean: 246369587 SD: ±92539193) and *grm5a*^*-/-*^ *grm5b*^*-/-*^ (N = 17, mean: 88194157 SD: ±54784433 animals expressing mbp:memGCaMP7s, reflecting baseline myelin Ca^2+^ activity (Mann–Whitney test p-value = <0.0001). Source data

To assess how mGluR5 signaling affects myelin Ca^2+^ activity we treated Tg(mbp:memGCamp7s) animals with CHPG or DMSO at 3 dpf and captured super-resolution confocal three-dimensional (3D) z-stacks by time-lapse microscopy at 1 Hz (per stack) for ~7 min immediately thereafter (Extended Data Fig. 7 and Fig. 4). We found that CHPG increased both the frequency and amplitude of Ca^2+^ transients compared to controls within 1 h of CHPG treatment, but that the effect of CHPG on transients had diminished 16 h following treatment (Extended Data Fig. 7b–i). Analysis of whole Tg(mbp:memGCamp7s) animals revealed that 16 h of treatment with the mGluR5 inhibitor MTEP, which reduced myelin sheath length also led to reduced myelin Ca^2+^, whereas 16 h of treatment with the agonist CHPG, which increased myelin sheath length increased overall myelin Ca^2+^ signal (Extended Data Fig. 8). Correspondingly, we saw reduced myelin Ca^2+^ signal in *grm5a*^−/−^*grm5b*^−^^/−^ mutants relative to controls. Together these data indicate that mGluR5 influences myelin Ca^2+^ in vivo.

To determine how mGluR5 activation affects the dynamics of myelin Ca^2+^ activity, we assessed whether the effects of CHPG treatment were mediated through endogenous mGluR5. We time-lapse imaged myelin Ca^2+^ in our *grm5a*^*−/−*^*grm5b*^−/−^ mutants treated with CHPG, and found that the stimulatory effect of CHPG seen in wild types on event amplitude was absent in animals lacking the target receptors (Fig. 4b′–f and Supplementary Videos 1–8). We did not recapitulate the effect of CHPG on event frequency in this separate set of experiments (Fig. 4d), suggesting that event amplitude is the most prominent aspect of myelin Ca^2+^ dynamics affected by mGluR5. We did not note any effect on event duration following CHPG treatment of controls or mutants (Fig. 4e). In summary, CHPG promotes an increase of high-amplitude myelin Ca^2+^ transients mediated by mGluR5 receptors. Together, our data indicate that activation of mGluR5 drives both high-amplitude Ca^2+^ transients in myelin sheaths and myelin sheath growth.

### Neuronal activity drives Ca^2+^ transients in myelin sheaths

Previous studies have indicated that neuronal activity can influence both myelin Ca^2+^ transients and sheath growth^37,40,53^. Here we wanted to test the hypothesis that mGluR5 mediates the effects of neuronal activity both on myelin Ca^2+^ and myelination.

To study the relationship between neuronal activity, myelin Ca^2+^, and myelination, we developed an all-optical imaging and opto-stimulation platform with which we could experimentally interrogate communication between active neurons and myelin. We reasoned that we would need to acutely regulate neuronal activity while imaging myelin Ca^2+^ transients, and also regulate activity for an extended period of time to assess the consequences on myelination. As a solution, we took an opto-stimulation approach to regulate neuronal activity, employing the red-shifted Channel-Rhodopsin ChRimsonR as an activator^62,63^. We expressed ChRimsonR in a set of descending premotor interneurons of the larval zebrafish (*chx10/V2a*(vsx2*)*-expressing interneurons) that project long axons into the spinal cord^64^, many of which are myelinated^40^. These neurons have well-defined roles in regulating complex motor behaviors of larval zebrafish, and their opto-stimulation induces physiologically relevant activity^64,65^.

**Fig. 5:**
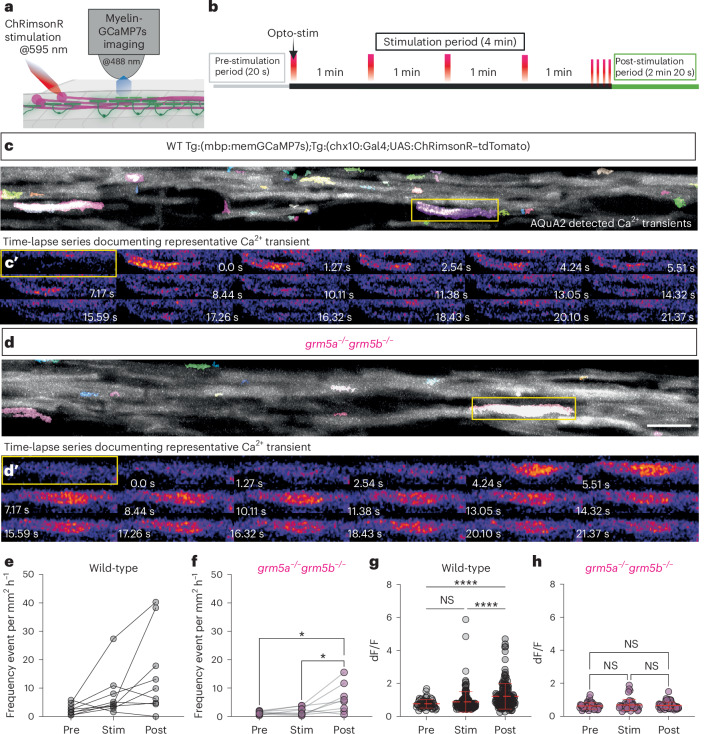
Opto-stimulation of neuronal activity induces mGluR5-dependent high-amplitude myelin Ca^2+^ transients. **a**, Opto-stimulation of ChRimsonR-expressing Chx10 interneurons is carried out during continuous time-lapse imaging of myelin Ca^2+^ transients in Tg(mbp:memGCaMP7s) animals. **b**, Optogenetic-stimulation paradigm, consisting of the pre-stimulation period ‘pre’ (gray), stimulation period ‘stim’ (black; single opto-stimulation/light pulse (red) per min, ending with 4× stimulations within 10 s) and post-stimulation period (green). **c**, Maximum projection of AQuA2-detected Ca^2+^ transients in a WT Tg(chx10:Gal4 UAS:ChRimsonR–tdTomato;mbp:memGCaMP7s) animal at 4 dpf. **c′**, Individual frames of time series showing the increase in fluorescence associated with myelin Ca^2+^ transient indicated by boxed area in **c**. **d**, Maximum projection of AQuA2-detected Ca^2+^ transients in a *grm5a*^*−/−*^*grm5b*^*−/−*^, Tg(chx10:Gal4 UAS:ChRimsonR–tdTomato; mbp:memGCaMP7s) animal at 4 dpf. Scale bar, 10 µm. **d′**, Individual frames of time series showing the increase in fluorescence associated with myelin Ca^2+^ transient indicated by boxed area in **d**. **e**, Frequency of myelin Ca^2+^ transients in WT animals across pre, stim and post-stim periods (two-sided Friedman test *P* = 0.0103; multiple comparisons test: pre versus stim *P* = 0.1017; pre versus post *P* = 0.0140; stim. versus post *P* > 0.9999) (WT *N* = 9, WT pre 2.55 ± 1.83, WT stim 7.51 ± 7.95; WT post 14.97 ± 14.75). **f**, Frequency of myelin Ca^2+^ transients per *grm5a*^*−/−*^*grm5b*^*−/−*^ animals over time across pre, stim and post-stim periods (two-sided Friedman test *P* = 0.008; multiple comparisons test: pre versus stim *P* > 0.9999; pre versus post: *P* = 0.0179; stim versus post *P* = 0.0373) (*grm5a*^*−/−*^*grm5b*^*−/−*^ *N* = 8, *grm5a*^*−/−*^*grm5b*^*−/−*^ pre 1.28 ± 0.53; *grm5a*^−/−^*grm5b*^−/−^ stim 1.93 ± 1.39; *grm5a*^−/−^*grm5b*^−/−^ post 6.34 ± 5.06). **g**, Individual WT Ca^2+^ amplitudes separated into three periods, pre (*n* = 65), stim (*n* = 175) and post-stimulation (*n* = 173) (One-way ANOVA, *P* = <0.0001, Kruskal–Wallis test multiple comparison: pre versus stim *P* > 0.9999; pre versus post *P* < 0.0001, stim versus post *P* < 0.0001) WT pre 0.76 ± 0.25, WT stim 0.88 ± 0.61 WT post 1.22 ± 0.78. **h**, Individual *grm5a*^−/−^*grm5b*^−/−^ Ca^2+^ amplitudes separated into the three periods, pre (*n* = 50), stim (*n* = 45) and post-stimulation (*n* = 45) (one-way ANOVA, Kruskal–Wallis test *P* *=* 0.6768, Dunn’s multiple comparison, pre versus stim *P* > 0.9999; pre versus post *P* > 0.9999, stim versus post *P* > 0.9999) *grm5a*^−/−^*grm5b*^−/−^ pre 0.63 ± 0.19, *grm5a*^−/−^*grm5b*^−/−^ stim 0.69 ± 0.34 *grm5a*^−/−^*grm5b*^−/−^ post 0.66 ± 0.20. Scale bar, 10 µm. Data are shown as mean ± s.d. Source data

**Extended Data Fig. 9:**
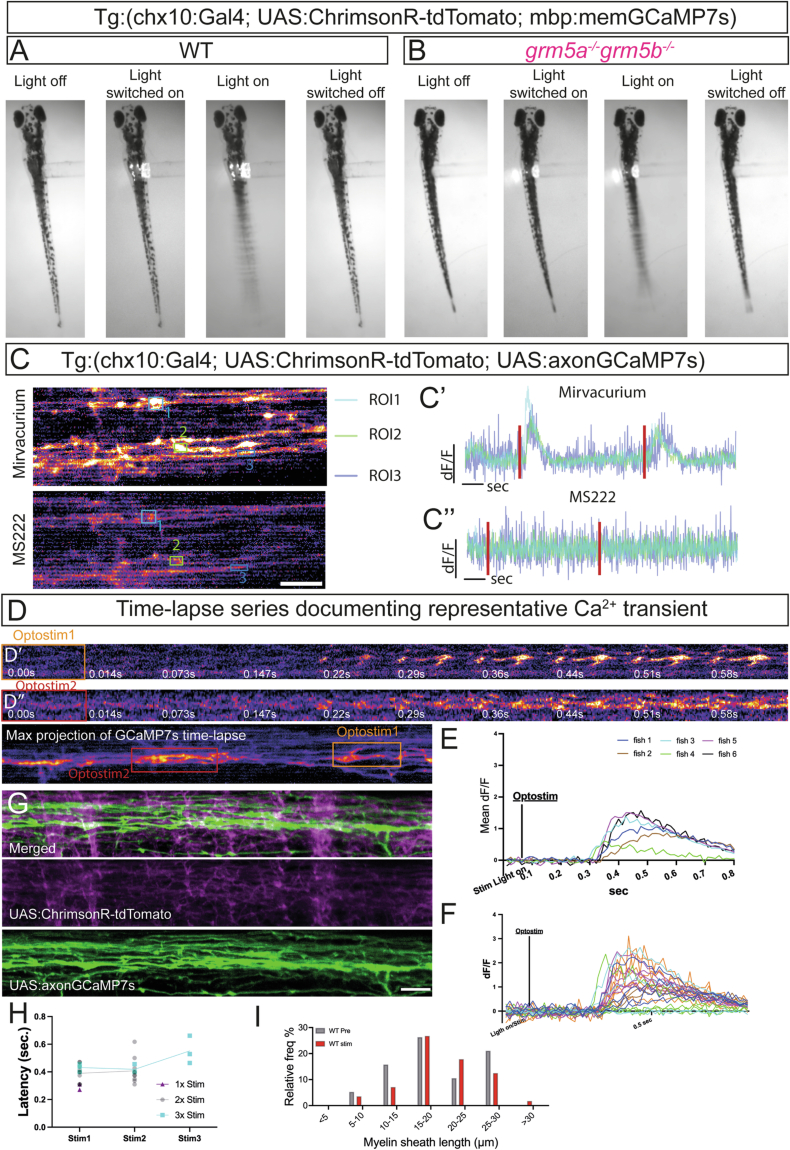
Opto-stimulation of WT and *grm5a*^*-/-*^*grm5b*^*-/-*^*chx10* interneurons. **A**: Opto-simulation of a WT Tg(chx10:Gal4;UAS:ChRimsonR-tdTomato) animal with a 595 nm fiber optic coupled laser induces a characteristic motor response. **B**: Opto-simulation of a *grm5a*^*-/-*^*grm5b*^*-/-*^ Tg(chx10:Gal4;UAS:ChRimsonR-tdTomato) animal with a 595 nm fiber optic coupled laser also induces a characteristic motor response. **C:** Images of chx10:Gal4;UAS:axonGCaMP7s in an animal treated with the neuromuscular blocker Mivacurium Chloride and subsequently with the Na+ channel blocker MS222. **C’:** dF/F trace of axonal GCaMP7s with Mivacurium, with opto-stimulations indicated by red lines. **C”:** dF/F trace of axonal GCaMP7s with MS222, with opto-stimulations indicated by red lines. **D**: Opto-stimulation of a WT Tg(chx10:Gal4;UAS:ChRimsonR-tdTomato;UAS:axonGCaMP7s) animal and corresponding time-lapse recording of axonal GCaMP7s signal following two separate stimulation steps (Optostim 1 (yellow) and Optostim2 (orange)) of two distinct ROIs. **E**: Mean dF/F traces of axonal GCaMP7s following two to three opto-stimulations per fish (N = 6). **F**: Individual dF/F traces of all axonal GCaMP7s following opto-stimulation of animals noted in E (n = 31). **G**: Representative image of the double transgene *Tg(chx10:Gal4;UAS:ChRimsonR-tdTomato;UAS:axonGCaMP7s)*. Scale bar, 10 µm. **H**: Latency between optical stimulation and max. amplitude dF/F of the axonal GCaMP7s signal after the first, second and third stimulation. **I**. Relative frequency of myelin sheaths exhibiting a myelin Ca^2+^ transient with and without opto-stimulation. Source data

**Extended Data Fig. 10:**
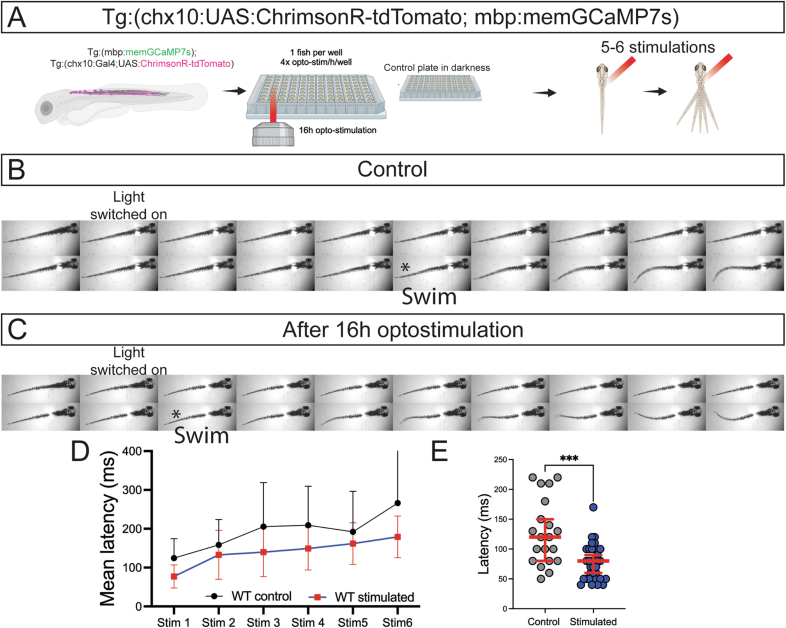
Characterization of motor responses to long-term (16 h) opto-stimulation. **A**: Long-term opto-stimulation of the chx10 pre-motor interneuron circuitry followed by assessment of motor response. The opto-stimulation was carried out identically to the experiments in Fig. 6. Control siblings were kept in a 96-well plate in the dark. After long-term opto-stimulation of chx10:ChRimsonR, the effects of acute activation were tested by assessing the latency between the onset of fiber illumination and initiation of the swimming movement. **B**: Optical stimulation of a control animal, showing induction of the swimming movement (asterisk). **C**: Optical stimulation of an animal following 16 h of long-term opto-stimulation, showing induction of swimming movement (asterisk). **D**: Mean latency between opto-stimulation and motor response in controls and animals that had undergone 16 h of long-term opto-stimulation. **E**: Latency from the first opto-stimulation to motor response per animal (Control N = 20, mean: 127.5 SD: ±54.86; Opto-stimulated N = 33, mean: 77.27, SD: ±29.71) (Mann–Whitney test; p-value = 0.0003). Source data

To stimulate ChRimsonR-expressing chx10 neurons of the brain while being able to carry out concomitant functional imaging, we used a 595-nm optical fiber, directed to the hindbrain (Methods and Fig. 5a). We first confirmed that opto-stimulation was sufficient to induce characteristic motor responses in control animals (Extended Data Fig. 9 and Supplementary Video 9). Notably, and in line with the grossly normal behavior of mutant animals, we found that opto-stimulation of chx10 neurons in *grm5a*^*−/−*^*grm5b*^*−/−*^ mutants also stimulated motor behavior, showing that we could recruit these neurons in animals with and without mGluR5 (Methods, Extended Data Fig. 9 and Supplementary Video 10). Notably, we found that our long-term (16 h) opto-stimulation paradigm did not lead to any observable habituation and that animals remained responsive to stimulation at the end of this period (Extended Data Fig. 10).

To be able to image Ca^2+^ activity during opto-stimulation we need to immobilize animals, while retaining neuronal excitability. Therefore, we used the neuromuscular blocking agent mivacurium chloride. We found that we were able to induce axon-localized GCaMP7s successfully upon opto-stimulation of chx10 neurons in animals treated with mivacurium chloride, but not in tricaine, as would be expected (Extended Data Fig. 9c,c′). This setup thus provided us with a system to manipulate neuronal activity and concomitantly assess myelin Ca^2+^ activity.

To assess the relationship between neuronal activity and myelin Ca^2+^, we combined our optical stimulation of chx10 neurons in the hindbrain with time-lapse recording of myelin Ca^2+^ activity in the spinal cord (Methods). We recorded myelin Ca^2+^ activity for 20 s before opto-stimulation, (referred to as ‘pre’) through four opto-stimulation steps of ~1–2 s separated by one minute and a fifth step of four repeated stimulations of ~1–2 s (referred to as ‘stim’). This was followed by continuous imaging of myelin Ca^2+^ for a further 2 min and 20 s (referred to as ‘post’) (Fig. 5b). We observed an increase in the frequency of myelin Ca^2+^ transients upon opto-stimulation of neuronal activity in control wild-type animals (Fig. 5c,e). Of note, we also observed an increase in myelin Ca^2+^ in *grm5a*^−/−^*grm5b*^−/−^ following opto-stimulation, indicating that mGluR5 does not regulate all aspects of activity-associated myelin Ca^2+^ activity (Fig. 5d,f and ‘Discussion’). We next assessed how opto-stimulation affected the kinetics of individual myelin Ca^2+^ transients and observed an increase in the amplitude of transients following the induction of neuronal activity (Fig. 5g). In this case, the effects of opto-stimulation on myelin Ca^2+^ were completely absent in mutants, with the amplitudes of events seen throughout the imaging protocol remaining the same over time in *grm5a*^−/−^*grm5b*^−/−^animals despite opto-stimulation (Fig. 5h). Together these data indicate that neuronal activity influences myelin Ca^2+^ transients in an mGluR5-dependent manner.

### Activity affects sheath length in an mGluR5-dependent manner

**Fig. 6:**
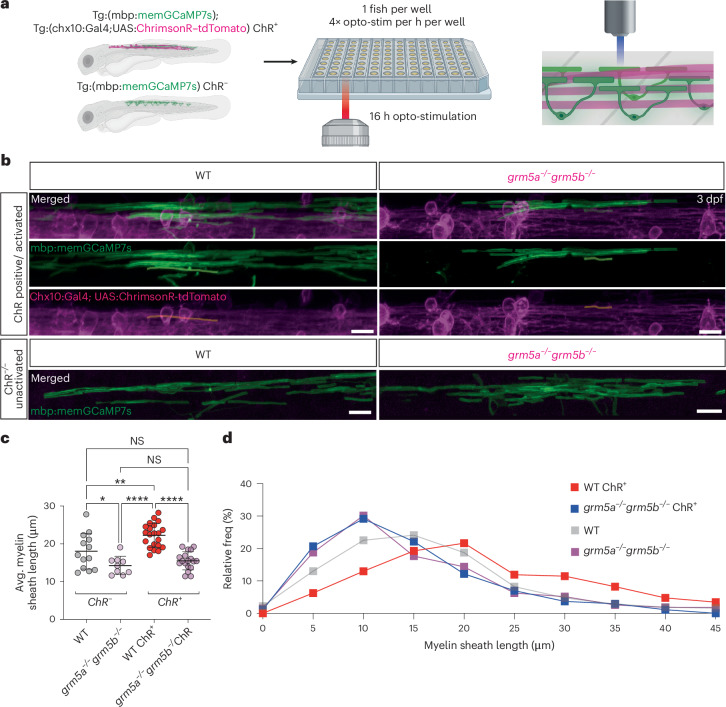
mGluR5 mediates myelin sheath growth in response to opto-stimulation of *chx10* interneurons. **a**, Experimental setup for long-term (16 h) opto-stimulation of individual ChRimsonR-positive and -negative Tg(mbp:memGCaMP7s) fish. Fish were screened for transgenesis, responsiveness to opto-stimulation and placed in individual wells of a 96-well plate and optically stimulated for 16 h before myelin morphology was assessed. **b**, Representative images, following 16 h of opto-stimulation, of a WT (left) and *grm5a*^−/−^*grm5b*^−/−^ mutant (right) expressing Tg(chx10:ChRimsonR–tdTomato;mbp:memGCaMP7s) (ChR^+^) (WT *N* = 21, *grm5a*^−/−^*grm5b*^−/−^ *N* = 18) (top). Representative images of Tg(mbp:memGCaMP7s), ChRimsonR–tdTomato negative (ChR^−^) WT (left) and *grm5a*^−/−^*grm5b*^−/−^ mutants (right) following 16 h of opto-stimulation (WT *N* = 13, *grm5a*^−/−^*grm5b*^−/−^ *N* = 9) (bottom). Scale bar, 10 µm. **c**, Avg. myelin sheath length per WT and *grm5a*^−/−^*grm5b*^−/−^ ChR^+^ and ChR^−^ animals (one-way ANOVA; *P* < 0.0001; Tukey’s multiple comparisons; WT ChR^+^ versus WT ChR^−^ *P* = 0.0032; WT ChR^+^ versus *grm5a*^−/−^*grm5b*^−/−^ ChR^+^ *P* < 0.0001; WT ChR^+^ versus *grm5a*^−/−^*grm5b*^−/−^ ChR^−^ *P* < 0.0001; WT ChR^−^ versus *grm5a*^−/−^*grm5b*^−/−^ ChR^+^ *P* = 0.1636; WT ChR^−^ versus *grm5a*^−/−^*grm5b*^−/−^ ChR^−^ *P* = 0.0488; *grm5a*^−/−^*grm5b*^−/−^ ChR^+^ versus *grm5a*^−/−^*grm5b*^−/−^ ChR^−^ *P* = 0.7810) WT ChR^−^ *N* = 13, 18.02 ± 4.73 WT ChR^+^ *N* = 21, 22.24 ± 3.16 *grm5a*^−/−^*grm5b*^−/−^ ChR^−^ *N* = 18, 14.24 ± 2.44 *grm5a*^−/−^*grm5b*^−/−^ ChR^+^ *N* = 18, 15.51 ± 2.40. **d**: Relative frequency distribution of individual sheath lengths, measured post-stimulation (WT ChR^+^ *n* = 462, 21.67 µm; WT ChR^−^ *n* = 315, 16.23 µm; *grm5a*^−/−^*grm5b*^−/−^ ChR^+^ *n* = 271, 14.04 µm; *grm5a*^−/−^*grm5b*^−/−^ ChR^−^ *n* = 271, 15.11 µm). Data are shown as mean ± s.d. Source data

To investigate how longer-term stimulation of neuronal activity affects myelination, we placed individual zebrafish into single wells of a 96-well plate and controlled the activity of ChRimson-expressing *chx10*-expressing neurons by widefield opto-stimulation (Methods). Before running each experiment, we first confirmed that individual chx10-ChRimsonR-expressing animals responded to opto-stimulation by eliciting characteristic swim responses. Once opto-stimulation was confirmed, we plated individual responders and ChRimson-negative controls into single wells (one animal per well) of a 96-well plate and carried out 16 h of opto-stimulation overnight from 2.5 dpf, light-activating animals every 15 min (Fig. 6a and Methods). To assess the effects of stimulating neuronal activity on myelination we imaged myelin using the stable transgenic reporter Tg(mbp:memGCaMP7s), which was expressed by both ChRimsonR-expressing and ChRimsonR-negative animals (Fig. 6). We found that 16 h of opto-stimulation of ChRimsonR-expressing wild types increased myelin sheath length at 3 dpf, in comparison to their non-ChRimsonR-expressing sibling controls (Fig. 6c). We next wanted to test whether this stimulatory effect of activity on myelin sheath length was present or absent in animals lacking mGluR5 activity. In contrast to the stimulation of wild-type controls, we found that opto-stimulation of ChRimsonR-expressing *grm5a*^−/−^*grm5b*^−/−^ had no effect on myelin sheath length, with both ChRimsonR-expressing and nonexpressing siblings exhibiting the reduced myelin sheath length characteristic of mutants (Fig. 6c,d). These findings indicate that mGluR5 mediates the effects of neuronal activity on myelin sheath elongation in vivo.

## Discussion

This study has identified a role for mGluR5 in mediating the effects of neuronal activity on myelination. We did not see an effect of mGluR5 inhibition on OPC or oligodendrocyte numbers, during early stages of myelination in zebrafish. Although we cannot rule out the possibility that mGluR5 affects early stages of oligodendrocyte lineage progression in other contexts, our findings define an unambiguous role for these receptors in bridging neuronal activity and myelination.

Our data identify a specific role for mGluR5 in both activity-dependent myelin Ca^2+^ signaling and sheath growth. Given that mGluR5 can localize to the growing tips of myelin sheaths, our results are consistent with a model in which localized release of glutamate from the axon^66,67^ activates mGluR5 on associated myelinating processes^37,68^; however, precisely how neuronal activity drives axonal release of glutamate, and how this activates mGluR5 on myelinating processes remains to be further characterized. For example, we need to understand how different rates of action potential firing along axons influence glutamate release, and how this in turn affects mGluR5, myelin Ca^2+^ and myelination. The kinetics of myelin Ca^2+^ transients occur over much longer timescales (seconds) than that of individual action potential firing (milliseconds). Therefore, it is possible that neurotransmitter-driven stimulation of myelinating processes requires trains of multiple action potentials to be fired within a certain time window. Alternatively, it is possible that the slow response of changes in myelin Ca^2+^ to action potential firing reflects the time required for metabotropic signaling. Although mGluR5 is required for activity-driven increases in myelin Ca^2+^ transient amplitude, we did note that neuronal activity can increase the frequency of myelin Ca^2+^ transients in the absence of mGluR5. This points to additional mechanisms by which action potential firing affects myelin Ca^2+^ responses, and likely also additional downstream responses and functions. Indeed, it is increasingly clear that there is a rich and diverse range of physiological interactions at the axon–myelin interface^69,70^. For example, a recent study has implicated the Kir4.1 inward rectifying potassium channel in influencing myelin Ca^2+^ and supporting the acute metabolic needs of highly active axons^71^. Previous studies in zebrafish have also shown a role for Ca^2+^ activity in the very early stages of sheath establishment and elongation^52,53^, with high-amplitude Ca^2+^ transients driving the retraction of nascent myelin sheaths^52^, possibly from inappropriate targets, during the selection of axons for myelination. The high-amplitude, long-duration events noted previously in nascent sheaths (~5 µm in length) in the process of being stabilized or not are distinct from the high-amplitude transients in established sheaths that we document here as being affected by mGluR5. Deconstructing the various ways in which neuronal activity influences localized signaling at the axon–myelin interface at distinct steps of myelin sheath formation, growth and maintenance, and function represent ongoing challenges for the field.

Our pharmacological and genetic studies point to a specific role for mGluR5 in regulating myelin sheath elongation, which is consistent with a model that different signals and receptors separately influence myelin sheath formation and stabilization and subsequent elongation along axons. Indeed, we and others have previously identified molecules in oligodendrocytes that affect sheath number without affecting sheath length^72,73^ and distinct effectors that influence sheath length without affecting sheath number^74,75^. However, here we found the mGluR5-driven increase in myelin sheath length, following expression in individual myelinating oligodendrocytes, was associated with a reduction in the number of sheaths made by those oligodendrocytes. This suggests the possibility that strong promotion of myelin sheath elongation might have the consequence of reducing the number of sheaths an individual oligodendrocyte can make or stabilize during the critical period of sheath formation^72^. This would suggest that individual oligodendrocytes may have a limited amount of myelin they can make over a certain time, for example during their dynamic early stages of myelination, and that this might require balancing myelin sheath number and size. This is consistent with observations that individual oligodendrocytes often make either a small number of large sheaths or a large number of shorter sheaths^76–79^. In the context of activity-regulated/adaptive myelination, it could be that neuronal activity can shift this balance. For example, highly active axons might stimulate robust elongation of myelin sheaths in a mGluR5-dependent manner, such that an individual oligodendrocyte cannot make or maintain a large number of sheaths. To completely understand how myelination by individual oligodendrocytes is influenced by neuronal activity, one will need models in which the number, length and thickness of sheaths can be accurately assessed as they change over time and upon distinct manipulations. Fully deconstructing the various ways in which activity can influence myelination will require the ability to control the distinct and defined neurons, axons and circuits while directly observing the dynamic behavior of oligodendrocytes, where their ability to respond to such activity is controlled in a cell type-specific and temporal manner.

Here we developed all-optical protocols to control neuronal activity and assess myelin Ca^2+^ signaling and fate. This provides capacity to further deconstruct molecular mechanisms linking neuronal activity and the responses of myelinating oligodendrocytes. In addition, our identification of mGluR5 as a key mediator of activity-regulated myelination will provoke assessment of the functional relevance of mGluR5-mediated myelination, in both zebrafish and other species, and in mature circuits as well as during development. One of the central tenets of adaptive myelination is that active changes to myelin might tune conduction velocities to optimize the timing of neural circuit function^4^. For example, it has been proposed that the pattern of myelination might be tuned to mediate highly specific conduction properties for the coordination of timing across neural circuits (for example, refs. ^80–83^). In the future, it will be important to investigate how mGluR5 function in oligodendrocytes influences myelination along axons with distinct modes of myelination, and to determine how alterations to myelin affect action potential conduction, the timing of neural circuit activity, and behavior. Activity-driven changes to myelination may have complementary, additional, or alternative consequences. For example, activity-regulated changes to myelination might influence axon domain formation and/or organization^9,83,84^, ion buffering^85,86^, glutamate synthesis^87^ and/or metabolic flux^28,71,88,89^, or even affect the likelihood of activity-driven ephaptic excitation of nearby axons^90^. Active-based regulation of myelination might also indirectly affect function, for example by affecting axonal transport^91^, which could influence the delivery of components to synapses to strengthen network-level connections. Through some or many of these processes, activity-regulated/adaptive myelination has the potential to greatly affect neural circuit function and the many behaviors that have been linked to dynamic changes to the oligodendrocyte lineage^11,15,25,73,89,92–100^, including associations first noted almost a century ago^101,102^.

Going forward, our discovery of mGluR5 as a mediator of activity-regulated myelination, and our development of a platform to identify further mediators of the effects of activity on myelinating oligodendrocytes, will help determine how the ongoing dynamic modulation of these cells affects axonal cell biology and physiology, through to system-wide function.

## Methods

### Zebrafish lines and maintenance

Adult zebrafish were housed at the University of Edinburgh Bioresearch and Veterinary Service zebrafish facility at the Queens Medical Research Institute. Studies were carried out with the approval of the UK home office and according to its regulations under the project license PP5258250. Adult animals were maintained at a 14-h day and 10-h night cycle. Embryos were maintained at 28.5 °C in 10 mM HEPES buffered E3-medium. In this study ‘Tg’ refers to stable transgenic lines, of which the following were used: Tg(mbp:memGCaMP7s), generated as part of this study, Tg(mbp:nls–eGFP)^103^, Tg(olig1:nls–mApple)^12^, Tg(chx10:Gal4;UAS:ChRimsonR–tdTomato)^63^ and Tg(UAS:axonGCaMP7s)^37^. All experiments were performed on larvae up to 7 dpf, before their sex could be determined.

### Pharmacological treatment of zebrafish

Zebrafish larvae were treated with compounds by immersion. The following compounds were used in this study: the mGluR5 antagonist MTEP 3-((2-methyl-1,3-thiazol-4-yl)ethynyl)pyridine; Tocris 1186195-60-7; 200 µM, 1% DMSO vehicle), the mGluR5 agonist CHPG (RS)-2-chloro-5-hydroxyphenylglycine (Tocris Cas:170846-74-9; 360 µM, 1% DMSO vehicle), the sodium channel blocker MS222/Tricaine (Sigma; 600 µM, no vehicle), the neuromuscular blocking agent mivacurium chloride (Abcam; 1.5 mg ml^−1^, no vehicle).

### Hybridization chain reaction RNA-FISH

The 5 dpf zebrafish larvae Tg(olig1:nls–mApple; olig1:memYFP or mbp:nls–eGFP; mbp:eGFP–CAAX) were fixed overnight in 4% paraformaldehyde at 4 °C. Larvae were washed 3 × 5 min in phosphate-buffered saline, then dehydrated and permeabilized in a series of methanol-washes (25% methanol/75% PBST, 50% methanol/50% PBST, 75% methanol/25% PBST, 2 × 100% methanol) for 5 min each at room temperature (RT). The final methanol-wash was replaced with fresh 100% methanol and larvae were incubated at −20 °C for ~2 h. Larvae were then rehydrated in a series of washes (75% methanol/25% PBST, 50% methanol/50% PBST, 25% methanol/75% PBST, 5 × 100% PBST) for 5 min each at RT.

Larvae were then incubated for 2 × 15 min in probe hybridization buffer (Molecular Instruments) at 37 °C, followed by incubation with 2 pmol (8 nM) of zebrafish *grm5a* or *grm5b* probes (Molecular Instruments) in probe hybridization buffer at 37 °C overnight. Excess probes were then removed by washing 4 × 15 min in probe wash buffer (Molecular Instruments) at 37 °C, followed by 3 × 5-min washes at RT in 5× SSCT buffer. Larvae were then incubated with amplification buffer (Molecular Instruments) for 30 min at RT, followed by incubation with 30 pmol hairpin h1 and 30 pmol of hairpin h2 (100 µM) in amplification buffer overnight at RT. Excess hairpins were removed by washing 6 × 10 min in 5× SSCT buffer at RT and 2 × 5 min in PBST.

Probes against *grm5a* and *grm5b* were provided by the manufacturer.

Imaging was performed on a Zeiss LSM 880 with Airyscan and a C-Apochromat ×63/1.2W Knorr UV-VIR-IR M27 objective.

### mGluR5 mutant generation and genotyping

The mGluR5 loss-of-function mutations disrupting zebrafish *grm5a* and *grm5b* were generated using CRISPR/Cas9. To generate mutant founders, we injected 2–3 nl of injection solution into fertilized eggs: 0.8 µl Spy Cas9 NLS (NEB, M0667), 0.8 µl gRNA targeting specific genes (details below, IDT), 0.5 µl Cas9 Buffer (NEB, B7203S) and 2.9 µl nuclease-free H_2_O.

The *grm5a* loss-of-function mutants were generated using a crRNA/gRNA targeting the sequence CTGCGAGGGCATGACCGTAC. The loss-of-function mutation for grm5a that was isolated and propagated for use in this study resulted in a 37-bp deletion that introduced a premature stop codon in exon 2 of the transcript. In addition, this gRNA targeted a restriction enzyme cut-site that we used to identify the mutations, as per the strategy of previous work^104^. For wild-type *grm5a*, the genotyping primers F 5′-GTAATGCTGGAGAGCAGAGCTT-3′ and R 5′-AGAGTTTCTTGCAGCTGCTCTT-3′ amplify a 249-bp product that is cut by HpyCHIII into 154-, 71- and 24-bp fragments. The delta_37-bp deletion abolishes one site, such that the PCR amplifies a 212-bp product and digestion yields 188 + 24-bp fragments.

*grm5b* mutants were generated using the same strategy, using a crRNA/gRNA targeting the sequence GCTGAGGTCCATACTGGTGG. The *grm5b* loss-of-function mutation used in this study resulted in a 28 bp deletion and introduction of an early stop codon in exon 1. To identify mutant alleles and animals we used the following primers: F 5′-ATAAGAGACTCCCTTGTGGCTG-3′ and R 5′-ACTATGTCCACCATGGCTCTG-3′ to amplify the *grm5b* locus. We digested the PCR product with the restriction enzyme BseNI (NEB: R0527S). For wild-type *grm5b*, the genotyping primers amplify a 290 bp product that is cut by BseNI into 196 and 94 bp. The delta_28bp deletion abolishes this site, such that the PCR amplifies a 262 bp product that is not cut by BseNI.

### Labeling and imaging individual oligodendrocytes

To mosaically label individual oligodendrocytes to assess their morphology, we injected fertilized eggs with plasmid encoding mbp:eGFP–CAAX, as described previously^76^. Before imaging, larvae were anesthetized with 600 μM tricaine in E3-medium and immobilized in low-melting-point agarose. Confocal z-stacks were obtained using a Zeiss LSM 880 microscope in Airyscan FAST super-resolution mode, using a ×20 water immersion lens (NA 1), and processed using the default Airyscan processing settings (Zen Black 2.3, Zeiss).

### Myelin thickness and nodal gap measurements

Myelin thickness and the size of nodal gaps were assessed using the Tg(mbp:memGCaMP7s) reporter in control and mutant animals at 3 and 7 dpf. Myelin thickness was assessed as per Bin et al.^105^ using super-resolution confocal fluorescent microscopy. We acquired super-resolution z-stacks with optimal z-step size using an LSM 880 Airyscan with a ×20 water dipping lens (NA 1). Myelin thickness was assessed along the Mauthner axon by measuring the diameter of the myelinated axon (a_x_), demarcated by the membrane-bound fluorescent reporter and by subtracting the diameter of the axon (b_x_) demarcated by the inner edges of the reporter. Three measurements were made per axon and an average taken, which was divided by 2 to denote myelin thickness: myelin thickness = (a_1_ − b_1_) + (a_2_ − b_2_) + (a_3_ − b_3_)/6).

Nodal gaps were identified as short gaps between adjacent myelin sheaths present in a single z-plane and the length between sheaths measured using the Fiji straight line tool (Fiji ImageJ v.1.51n). All images were assessed blinded to the genotype.

### High-content imaging of OPC and oligodendrocyte populations

To quantify OPC and oligodendrocyte number we used the transgenic reporters Tg(olig1:nls–mApple) and Tg(mbp:nls–eGFP), respectively. We imaged individual transgenic animals using the previously described Vertebrated-Automated-Screening-Technology (VAST)-spinning disc confocal microscope (SDCM) system, which allows automated delivery of zebrafish larvae from the wells of a 96-well plate to a capillary for automated orientation and high-speed imaging. We imaged animals using the SDCM (Zeiss) using a ×10 water dipping lens (NA 0.5), collecting six-tiled arrays of confocal z-stacks that captured the entire spinal cord of larvae. Individual z-stacks were stitched using customized scripts^103^ and cell numbers were quantified using the ‘cell counter’ function of Fiji.

### Cloning of zebrafish *grm5a* and *grm5b* cDNA

To clone wild-type *grm5a* and *grm5b* complementary DNA we carried out PCR with high-fidelity DNA polymerase Phusion (New England Biolabs) from a pool of wild-type zebrafish total cDNA (reverse-transcribed from total mRNA extracted from 5 dpf zebrafish of the wild-type AB strain). For *grm5a*, we used forward primer 5′-ATG GGG GGT TTT CAT TTG CTG G-3′ (predicted start codon underlined) and reverse primer 5′-TCA GAG CGA AGA CGA GCT CTG G-3′ (predicted stop codon underlined). For *grm5b*, we used forward primer 5′-ATG GTC ATT TTG TGT TCT CTC G-3′ (predicted start codon underlined) and reverse primer 5′-CCG TGC CGG TCC TCA TAA T-3′ (predicted stop codon underlined). These PCRs amplified approximately 3.6-kbp products, which we purified and TOPO-cloned (using the zero Blunt TOPO PCR Cloning kit, Thermo Fisher Scientific) to generate pCRII–grm5a and pCRII–grm5b. We sequenced multiple clones and identified 3,522-bp and 3,588-bp sequences that matched the predicted coding sequences (Ensembl entries ENSDART00000193829.1 and ENSDART00000170398.2).

### Generation of *grm5a* and *grm5b* expression constructs

To tag *grm5a* and *grm5b* sequences at the C terminus with a GGGGS linker followed by monomeric eGFP, and simultaneously produce tol2kit-compatible 3′-entry vectors, we used recombinant PCR with Phusion polymerase. For attB2-grm5a-linker we used primers 5′-GGG GAC AGC TTT CTT GTA CAA AGT GGG CCA CCA TGG GGG GTT TTC ATT TGC TGG TG-3′ (start codon underlined) and 5′-GGA CCC TCC GCC TCC GAG CGA AGA CGA GCT-3′ (linker-sequence underlined). For attB2-grm5b-linker we used primers 5′-GGG GAC AGC TTT CTT GTA CAA AGT GGG CCA CCA TGG TCA TTT TGT GTT CTC TCG C-3′ (start codon underlined) and 5′-GGA CCC TCC GCC TCC TAA TGA AGA CGA GCT-3′ (linker-sequence underlined). For the linker–meGFP–polyA-attB3R sequence common to both, we used primers 5′-GGA GGC GGA GGG TCC GTG AGC AAG GGC GAG-3′ (linker-sequence underlined) and 5′-GGGGACAACTTTGTATAATAAAGTTGAAAAAACCTCCCACACCTCCCCCTG-3′ (polyA-sequence underlined). We purified these primary PCR products and used equimolar amounts as template for the second recombinant PCR, using only the attB-containing primers.

We then used BP Clonase II to recombine the complete PCR products with pDONRP2R-P3 (plasmid #220 from the tol2kit), to generate Gateway-compatible 3′ entry vectors p3E-grm5a/grm5b–eGFP, which we verified by Sanger sequencing.

These entry vectors were used in combination with 5E–mCherry–CAAX (in inverted orientation) and ME–10xJanus to generate the Janus-configuration bicistronic expression vector pTol2–mCherry–CAAX–10xJanus–grm5a/b–eGFP.

These entry vectors were also used in combination with 5E–mbp and ME–mem–mScarlet-2A to generate constructs mbp:mem–mScarlet-2A–grm5a–eGFP and mbp:mem–mScarlet-2A–grm5b–eGFP.

### Live imaging of myelin Ca^2+^ activity

To assess myelin Ca^2+^ activity we generated a new transgenic line in which the genetically encoded Ca^2+^ indicator GCaMP7s was tethered to the membrane and expressed in myelinating oligodendrocytes under the control of the mbp promoter sequence^76^. To generate the mbp:memGCaMP7s; cryaa:mCherry construct, wherein cryaa:mCherry serves as an independent marker of transgenesis, we first created a tol2kit-compatible middle-entry vector containing the coding sequence for the membrane-tethered memGCaMP7s, pME-memGCaMP7s. To do this, we digested our previously generated plasmid pME-jGCaMP7s^37^ (containing an untethered, cytoplasmic GCaMP7s coding sequence) at the start codon with NcoI-HF enzyme (GCCACC**ATG**G, NcoI recognition-sequence underlined, start codon in bold, NcoI-HF from New England Biolabs). Into this digested vector we then ligated two annealed primers, 5′-CATGGGCTGTGTGCAATGTAAGGATAAAGAAGCAACAAAACTGACGGG-3′ and 5′-CATGCCCGTCAGTTTTGTTGCTTCTTTATCCTTACATTGCACACAGCC-3′, phosphorylated at the 5′ end (from IDT DNA Technologies), which encode the myristoylation motif of human Fyn kinase flanked by overhanging NcoI-compatible ends. The sequence for this plasmid was verified by Sanger sequencing.

To generate the final Tol2 expression construct (mbp:memGCaMP7s; cryaa:mCherry), we then recombined 10fmol of the following entry vectors: previously described 5′-entry vector 5E-mbp^76^, ME-memGCaMP7s and 3E-polyA from the tol2kit^106^; and 20fmol of destination vector pDestTol2pA2-cryaa:mCherry^107^ (Addgene #64023), using LR-Clonase II Plus. Then, 3–4 clones were tested for correct recombination by restriction enzyme digestion.

To establish a stable transgenic line, we injected 5 pg of Tg(mbp:memGCaMP7s; cryaa:mCherry) plasmid DNA with 50 pg *tol2* transposase mRNA into wild-type zebrafish eggs at the one-cell stage. This yielded memGCaMP7s-expressing oligodendrocytes in injected embryos, which were then raised to adulthood. Founders were identified by screening their F1 offspring for germline transmission using the cryaa:mCherry marker.

To acquire images of myelin Ca^2+^ in Tg(mbp:memGCaMP7s) animals, we performed time-lapse imaging on a Zeiss LSM 880 Airyscan equipped using a ×20 water dipping objective (NA 1). To best resolve individual myelin sheaths we imaged the dorsal spinal cord in all animals. Time-lapse recordings were made in Airyscan FAST super-resolution mode with ×3.5 digital zoom at around 1 Hz with individual 3D confocal stacks of ten z-planes acquired at each time-point. Time-lapse datasets were processed using Zen Pro Black, with resulting czi time-lapse images registered using the turbo-reg function in Fiji, before saving in tif format for analysis using AQuA2.

### AQuA2 to quantify myelin sheath Ca^2+^ activity

The complex patterns of Ca^2+^ activity within myelin sheaths prompted us to explore event-based quantification methods with AQuA^108^; however, AQuA yielded suboptimal results, generating a large number of false positives due to the low signal-to-noise ratio and the texture of myelin sheath imaging. To address these and other issues relating to the analysis of functional imaging data, we employed AQuA2, an improved version of AQuA (see ref. ^61^ for extensive details). In brief, AQuA2 builds upon the core concept of AQuA, which involves the direct detection of signal events from dF, rather than identifying the region of interest. In contrast to AQuA’s approach of clustering voxels into events, which is vulnerable to local fluctuations, AQuA2 introduces a pipeline based on spatiotemporal segmentation. Utilizing information from a broader range of surrounding pixels, harnessing machine learning techniques, and applying advanced statistical testing, AQuA2 exhibits enhanced robustness and accuracy. With pre-set parameters designed for myelin sheath Ca^2+^ data, AQuA2 reliably captures Ca^2+^ transients and provides automated quantification of a range of parameters^61^.

### Optogenetic-based stimulation of *chx10* interneurons

To activate neurons, we employed the red-shifted Channel-Rhodopsin ChRimsonR, and the previously generated transgenic line Tg(chx10:Gal4; UAS-ChRimsonR–tdTomato)^63^ to activate Chx10-expressing interneurons known to drive specific swimming behaviors. To identify animals that express ChRimsonR and respond to opto-stimulation, we first screened zebrafish larvae at 4 dpf for expression of ChRimsonR–tdTomato and then tested the response to opto-stimulation by illuminating animals placed in the wells of a 96-well plate via a short pulse of 595 nm light at 1–3 µW using widefield illumination at ×10 magnification (Zeiss Observer Z1). Successful responders were identified by the induction of characteristic swimming driven by Chx10 neurons. Successful responders were used either to test how acute increases in neuronal activity affected myelin Ca^2+^ or longer-term increases affected myelination. We used this opto-stimulation setup together with high-speed imaging of the motor response to assess if ChRimsonR-expressing animals retain their excitability after 16 h of opto-stimulation. Animals were opto-stimulated for 16 h as described above and then mounted in a Petri dish in low-melting point agarose droplets. After the agarose was solidified, the fish’s tail was freed by carefully removing the agarose. The Petri dish was then filled with embryo medium containing the head-restrained animal and placed on an illumination stage. After 10–15 min of recovery, an optical fiber (Thorlabs, M595F2; CFML12L10; M89L01; output power, 140 mW) was positioned next to the hindbrain of the animal. The optical stimulation of the ChRimsonR-expressing chx10 interneurons was recorded at 100 frames per second using an Orca Flash4 camera. The latency between opto-stimulation (onset of light emission from the fiber) and initiation of body movement was then measured manually using Fiji.

### Opto-stimulation coupled to imaging of myelin Ca^2+^

To test whether neuronal activity could affect myelin Ca^2+^ we stimulated neurons in Tg(chx10:Gal4; UAS-ChRimsonR–tdTomato; mbp:memGCaMP7s) animals using an LED (M595F2, Thorlabs), with light delivered to the hindbrain via an optical fiber (Thorlabs, M64L01) that was coupled to a 0.22 NA cannula (Thorlabs, CFMLC21L20). Before functional imaging, we carried out a second confirmation that individual pre-screened animals were responsive to opto-stimulation, by inducing swimming behaviors in animals that were head-restrained in low-melting point agarose, but with trunk and tail regions free to move. Successful responders were then treated with the neuromuscular blocking agent mivacurium chloride (1.5 mg ml^−1^) to prevent movement following opto-stimulation during functional imaging. For live imaging, individual animals were re-mounted in low- melting point agarose in a lateral orientation, with time-lapse imaging carried out as previously noted.

### Opto-stimulation and assessment of myelination

To carry out longer-term activation of neurons, we opto-stimulated Tg(chx10:Gal4; UAS-ChRimsonR–tdTomato; mbp:memGCaMP7s) from ~2.5 dpf to 3 dpf for 16 h, before assessing myelination by confocal live imaging.

For long-term opto-stimulation, individual fish selected for transgene expression and exhibiting swim-based responsiveness to opto-stimulation were re-plated into individual wells of a 96-well plate. Control animals not expressing ChRimsonR opsin were also plated in an interspersed manner into wells of the same 96-well plate and subject to same opto-stimulation paradigm. To induce long-term stimulation, animals were illuminated using a fully motorized Zeiss Observer Z1 system, whereby the microscope automatically moved from well to well, stimulating each fish every 15 min over the duration of 16 h, resulting in 64 individual opto-stimulations steps per animal. During the stimulation the fish were kept at 28.5 °C and the plate was sealed to avoid any contaminating light-based illumination. After 16 h of stimulation myelination was assessed by imaging Tg(mbp:memGCaMP7s) using an LSM 880 (Zeiss). For live imaging of myelin, individual fish were anesthetized using MS222, mounted laterally in low-melting point agarose and imaged using Airyscan super-resolution mode with a ×20 water dipping lens (NA 1).

### Analyses of free-swimming behavior

To test if mutations disrupting *grm5a* and or *grm5b* had any obvious effect on the general activity of the zebrafish larvae, we assessed their swimming behavior.

We assessed free-swimming behavior of larvae at 5 dpf, using a fully equipped Zantiks MWP unit (Zantiks), which allows induction of well-characterized swimming responses to alternating patterns of light and dark illumination^109^. We collected zebrafish from crosses of *grm5a*^*+/*^^−^*grm5b*^*+/−*^ parents and placed them individually into single wells of a 96-well plate. We then recorded their swimming distance (in mm) over the course of 1 h at 28.5 °C. The fish were first allowed to acclimatize to the environment of 28 °C for 20 min, before being tracked further for 1 h, which was composed of exposure to three cycles of 10-min darkness followed by 10-min light illumination. The recorded swimming distance during the acclimatization phase was discarded and only behavior during the post-acclimation dark and light cycles were used for analysis. Following recording, animals were genotyped for mutations in *grm5a* and *grm5b*, as noted above.

### Experimental design and randomization

Each experiment was repeated using multiple clutches of fish on multiple days. Files containing data that had to be analyzed manually were codified in a randomized manner to allow blinded analysis, or samples were genotyped after analyses, ensuring blinding to experimental group. GCaMP7s time-lapse data were not blinded to treatment group, but analyses were carried out in an automated and thus unbiased manner using pre-set parameters integral to AQuA2. Recorded datafiles of insufficient quality were excluded from the analysis.

### Statistics and reproducibility

Data were organized using Microsoft Excel 365. Statistical analyses were carried out using Graph Pad Prism (v.8–10, up to 10.1.2). Data were tested for normality before being analyzed accordingly. All individual data points are indicated throughout alongside demarcation of means and s.d. or medians and interquartile ranges. For statistical analyze *N* represents a single fish, with the corresponding datapoint reflecting either mean or median of all the measurements. Illustration of parameters not depicted per animal are denoted as *n*. All in vivo experiments were conducted on multiple biological replicates (more than three). No statistical methods were used to predetermine sample sizes but our sample sizes are similar to those reported in previous publications^52^

### Reporting summary

Further information on research design is available in the Nature Portfolio Reporting Summary linked to this article.

## Online content

Any methods, additional references, Nature Portfolio reporting summaries, source data, extended data, supplementary information, acknowledgements, peer review information; details of author contributions and competing interests; and statements of data and code availability are available at 10.1038/s41593-025-01956-9.

## Supplementary information

Reporting Summary

Supplementary Video 1 Video showing myelin Ca^2+^ transient (raw data) in DMSO-treated wild-type animal.

Supplementary Video 2 Video showing myelin Ca^2+^ transient (AQuA2 output) in DMSO-treated wild-type animal.

Supplementary Video 3 Video showing myelin Ca^2+^ transient (raw data) in CHPG-treated wild-type animal.

Supplementary Video 4 Video showing myelin Ca^2+^ transient (AQuA2 output) in CHPG-treated wild-type animal.

Supplementary Video 5 Video showing myelin Ca^2+^ transient (raw data) in DMSO-treated in grm5a^−/−^, grm5b^−/−^ mutant animal.

Supplementary Video 6 Video showing myelin Ca^2+^ transient (AQuA2 output) in DMSO-treated in grm5a^−/−^, grm5b^−/−^ mutant animal.

Supplementary Video 7 Video showing myelin Ca^2+^ transient (raw data) in CHPG-treated grm5a^−/−^, grm5b^−/−^ mutant animal.

Supplementary Video 8 Video showing myelin Ca^2+^ transient (AQuA2 output) in CHPG-treated grm5a^−/−^, grm5b^−/−^ mutant animal.

Supplementary Video 9 Opto-stimulation of chx10 neuron-driven motor activity in wild-type animal.

Supplementary Video 10 Opto-stimulation of chx10 neuron-driven motor activity in grm5a^−/−^, grm5b^−/−^ mutant animal.

## Source data

Source Data Statistical source data. Raw measurements and values for Figs. 1–6 and Extended Data Figs. 1–3 and 5–10.

## Data Availability

The plasmids generated in this study, mbp:mem–mScarlet-P2A–grm5a–eGFP, mbp:mem–mScarlet-P2A-grm5b–eGFP, 10xUAS:mem–mScarlet-P2A–grm5a–eGFP, 10xUAS:mem–mScarlet-P2A–grm5b–eGFP will be made available upon request. The following zebrafish lines, generated for this study, will be made available on request: Tg(mbp:memGCaMP7s), Tg(chx10:Gal4;UAS:ChRimsonR–tdTomato; mbp:memGCaMP7s) as well as *grm5a*, *grm5b* mutant fish. Any additional information required to reanalyze the data reported in this paper is available from the lead contact upon request. Source data are provided with this paper.
