# Supplementary material for: Activity-driven myelin sheath growth is mediated by mGluR5
Source: Nat Neurosci. 2025 May 14;28(6):1213–25. doi: 10.1038/s41593-025-01956-9 (PMC12148935; doi:10.1038/s41593-025-01956-9)
Supplement: Supplementary file 1 — Reporting Summary [file 41593_2025_1956_MOESM1_ESM.pdf]

nature portfolio

Corresponding author(s): David Lyons

Last updated by author(s): Mar 11, 2025

Reporting Summary

Nature Portfolio wishes to improve the reproducibility of the work that we publish. This form provides structure for consistency and transparency in reporting. For further information on Nature Portfolio policies, see our Editorial Policies and the Editorial Policy Checklist.

Statistics

For all statistical analyses, confirm that the following items are present in the figure legend, table legend, main text, or Methods section.

n/a	Confirmed	
☐	☒	The exact sample size (*n*) for each experimental group/condition, given as a discrete number and unit of measurement
☐	☒	A statement on whether measurements were taken from distinct samples or whether the same sample was measured repeatedly
☐	☒	The statistical test(s) used AND whether they are one- or two-sided<br>*Only common tests should be described solely by name; describe more complex techniques in the Methods section.*
☒	☐	A description of all covariates tested
☐	☒	A description of any assumptions or corrections, such as tests of normality and adjustment for multiple comparisons
☐	☒	A full description of the statistical parameters including central tendency (e.g. means) or other basic estimates (e.g. regression coefficient) AND variation (e.g. standard deviation) or associated estimates of uncertainty (e.g. confidence intervals)
☐	☒	For null hypothesis testing, the test statistic (e.g. F, t, r) with confidence intervals, effect sizes, degrees of freedom and P value noted<br>*Give P values as exact values whenever suitable.*
☒	☐	For Bayesian analysis, information on the choice of priors and Markov chain Monte Carlo settings
☒	☐	For hierarchical and complex designs, identification of the appropriate level for tests and full reporting of outcomes
☒	☐	Estimates of effect sizes (e.g. Cohen's *d*, Pearson's *r*), indicating how they were calculated

Our web collection on statistics for biologists contains articles on many of the points above.

Software and code

Policy information about availability of computer code

Data collection	Zen Black 2.3 (Zeiss)
Data analysis	Fiji Image J v1.51n, GraphPad Prism versions 8-10 (up to version 10.1.2), Adobe Illustrator 2020 (24.0.2), Microsoft Excel 365, AQuA2.

For manuscripts utilizing custom algorithms or software that are central to the research but not yet described in published literature, software must be made available to editors and reviewers. We strongly encourage code deposition in a community repository (e.g. GitHub). See the Nature Portfolio guidelines for submitting code & software for further information.

Data

Policy information about availability of data

All manuscripts must include a data availability statement. This statement should provide the following information, where applicable:

- Accession codes, unique identifiers, or web links for publicly available datasets
- A description of any restrictions on data availability
- For clinical datasets or third party data, please ensure that the statement adheres to our policy

All data supporting the findings of this study are available within the paper and its Supplementary Information.

Research involving human participants, their data, or biological material

Policy information about studies with human participants or human data. See also policy information about sex, gender (identity/presentation), and sexual orientation and race, ethnicity and racism.

Reporting on sex and gender	No human patients, their data, or biological material were used in this study.
Reporting on race, ethnicity, or other socially relevant groupings	No human patients, their data, or biological material were used in this study.
Population characteristics	See above
Recruitment	NA
Ethics oversight	NA

Note that full information on the approval of the study protocol must also be provided in the manuscript.

Field-specific reporting

Please select the one below that is the best fit for your research. If you are not sure, read the appropriate sections before making your selection.

☒ Life sciences ☐ Behavioural & social sciences ☐ Ecological, evolutionary & environmental sciences

For a reference copy of the document with all sections, see nature.com/documents/nr-reporting-summary-flat.pdf

Life sciences study design

All studies must disclose on these points even when the disclosure is negative.

Sample size	This was the first study to look at receptor specific axon myelin communication in zebrafish; therefore it was not possible to do power calculations prior to the study. No statistical methods were used to pre-determine sample size, but our sample sizes are similar to those reported in previous publications (Marshall-Phelps et al 2020, Almeida et al 2023) and was also based on including fish from multiple clutches of animals. Sample sizes for each experiments are included in the figure legends.
Data exclusions	Timelapse images displaying large drifts were excluded, as well as Ca2+ transients which were leaving the field of view and such that originate from outside the field of view.
Replication	All analysis were performed on animals from at least three different clutches, with all conclusions replicated across independent experiments.
Randomization	Randomization was applied for analysing individual oligodendrocyte morphology. Either the files name/treatment group or the genotype of the animals was blinded.
Blinding	All manual analyses were carried out blinded to experimental group. AQuA2 is an. automated analysis pipeline that does not require blinding.

Reporting for specific materials, systems and methods

We require information from authors about some types of materials, experimental systems and methods used in many studies. Here, indicate whether each material, system or method listed is relevant to your study. If you are not sure if a list item applies to your research, read the appropriate section before selecting a response.

Materials & experimental systems

n/a	Involved in the study	
☒	☐	Antibodies
☒	☐	Eukaryotic cell lines
☒	☐	Palaeontology and archaeology
☐	☒	Animals and other organisms
☒	☐	Clinical data
☒	☐	Dual use research of concern
☒	☐	Plants

Methods

n/a	Involved in the study	
☒	☐	ChIP-seq
☒	☐	Flow cytometry
☒	☐	MRI-based neuroimaging

Animals and other research organisms

Policy information about studies involving animals; ARRIVE guidelines recommended for reporting animal research, and Sex and Gender in Research

Laboratory animals	Zebrafish (danio rerio) up to 4 days post-fertilization in age. mGluR5 mutant grm5a grm5b and grm5a-grm5b. Transgenic lines used: Tg(mbp:memGCaMP7s), Tg(olig1:nls-mApple) , Tg(UAS:axonGCaMP7s), Tg:(mbp:nls-eGFP), Tg(chx10:Gal4;UAS:ChrimsonR-tdTomato).
Wild animals	No wild animals were used in this study.
Reporting on sex	Zebrafish (danio rerio) were used for experiments before the onset of sexual differentiation.
Field-collected samples	No field-collected samples were used in this study.
Ethics oversight	Adult zebrafish were housed at the University of Edinburgh Bioresearch and Veterinary Service zebrafish facility at the Queens Medical Research Institute. Studies were carried out with the approval of the UK home office and according to its regulations under the project licence PP5258250.

Note that full information on the approval of the study protocol must also be provided in the manuscript.

Plants

Seed stocks	NA
Novel plant genotypes	NA
Authentication	NA
